# Clinical *Staphylococcus aureus* inhibits human T-cell activity through interaction with the PD-1 receptor

**DOI:** 10.1128/mbio.01349-23

**Published:** 2023-10-05

**Authors:** Maiken Mellergaard, Sarah Line Skovbakke, Stine Dam Jepsen, Nafsika Panagiotopoulou, Amalie Bøge Rud Hansen, Weihua Tian, Astrid Lund, Rikke Illum Høgh, Sofie Hedlund Møller, Romain Guérillot, Ashleigh S. Hayes, Lise Tornvig Erikstrup, Lars Andresen, Anton Y. Peleg, Anders Rhod Larsen, Timothy P. Stinear, Aase Handberg, Christian Erikstrup, Benjamin P. Howden, Steffen Goletz, Dorte Frees, Søren Skov

**Affiliations:** 1 Department of Veterinary and Animal Sciences, Laboratory of immunology, Section for Preclinical Disease Biology, Faculty of Health and Medical Sciences, University of Copenhagen, Copenhagen, Denmark; 2 Biotherapeutic Glycoengineering and Immunology, DTU Bioengineering, Department of Biotechnology and Biomedicine, Technical University of Denmark, Lyngby, Denmark; 3 Department of Microbiology and Immunology, University of Melbourne, at the Peter Doherty Institute for Infection and Immunity, Melbourne, Victoria, Australia; 4 Department of Clinical Microbiology, Aarhus University Hospital, Aarhus, Denmark; 5 Department of Microbiology, Monash University, Melbourne, Victoria, Australia; 6 Department of Microbiology, Infection Program, Monash Biomedicine Discovery Institute, Monash University, Melbourne, Victoria, Australia; 7 Centre to Impact Antimicrobial Resistance, Monash University, Melbourne, Victoria, Australia; 8 Statens Serum Institute, Microbiology and Infection Control, Copenhagen, Denmark; 9 Department of Clinical Biochemistry, Aalborg University Hospital, North Denmark Region, Aalborg, Denmark; 10 Department of Clinical Medicine, Aalborg University, Aalborg, Denmark; 11 Department of Clinical Immunology, Aarhus University Hospital, Aarhus, Denmark; 12 Food Safety and Zoonosis, Department of Veterinary and Animal Sciences, Faculty of Health and Medical Sciences, University of Copenhagen, Copenhagen, Denmark; Harvard Medical School, Boston, Massachusetts, USA; University of Nebraska Medical Center, Omaha, Nebraska, USA

**Keywords:** clinical *Staphylococcus aureus*, immune evasion, adaptive immunity, ClpP mutation, T cells

## Abstract

*Staphylococcus aureus* (*S. aureus*) represents a major clinical challenge due to its explicit capacity to select mutations that increase antibiotic resistance and immune evasion. However, the molecular mechanisms are poorly defined, especially for adaptive immunity. Cancer immunotherapy targeting programmed cell death protein 1 (PD-1) enhances T-cell activity and is emerging for the treatment of certain viral infections, while its potential against bacterial infections remains elusive. We show that an *S. aureus clpP* mutant, selected during clinical antibiotic therapy, inhibits T-cell activity by directly interacting with PD-1 on human T cells. Specificity of the interaction was confirmed using recombinant PD-1, as well as PD-1 overexpressing and knock out cells. Moreover, the PD-1-binding *S. aureus* inhibited intracellular calcium mobilization, T-cell proliferation, CD25 expression, and IL-2 secretion, while the key effects were alleviated by antibody-mediated PD-1 blockade using an engineered IgG1-based anti-PD-1 antibody. Our results suggest that *clpP* mutant *S. aureus* directly targets PD-1 to evade immune activation and that therapeutic targeting of PD-1 may be used against certain staphylococcal infections.

**IMPORTANCE:**

Therapies that target and aid the host immune defense to repel cancer cells or invading pathogens are rapidly emerging. Antibiotic resistance is among the largest threats to human health globally. *Staphylococcus aureus* (*S. aureus*) is the most common bacterial infection, and it poses a challenge to the healthcare system due to its significant ability to develop resistance toward current available therapies. In long-term infections, *S. aureus* further adapt to avoid clearance by the host immune defense. In this study, we discover a new interaction that allows *S. aureus* to avoid elimination by the immune system, which likely supports its persistence in the host. Moreover, we find that blocking the specific receptor (PD-1) using antibodies significantly relieves the *S. aureus*-imposed inhibition. Our findings suggest that therapeutically targeting PD-1 is a possible future strategy for treating certain antibiotic-resistant staphylococcal infections.

## INTRODUCTION

*Staphylococcus aureus* (*S. aureus*) confers asymptomatic, persistent colonization of 30–40% of the human population, but can also cause a range of infections (1, 2). Especially, skin and soft tissue infections are common and often associate with the development of more severe infections, including bacteremia and endocarditis (3). New therapies are needed due to the intrinsic ability of *S. aureus* to evade the immune system along with the development of antibiotic resistance (4 – 6).

T-cell responses are vital for *S. aureus* control (7). Individuals born with T-cell deficiencies in the T_H_17 response are highly susceptible to *S. aureus* infections, as are HIV-infected patients with reduced CD4^+^ T-cell function (8 – 10). *S. aureus*-specific T cells in healthy individuals can reach up to 5% of the peripheral T-cell pool (11). Several genetic lineages of *S. aureus* secrete highly potent superantigens that cause severe CD4^+^ T-cell activation, inflammation, and subsequent T-cell desensitization through direct interaction with specific Vβ-domains of the T-cell receptor (TCR) (12). T cells are tightly regulated by immune checkpoint receptors, including programmed cell death protein 1 (PD-1), and checkpoint inhibitors in the form of specific blocking antibodies against PD-1, and its ligands have shown remarkable clinical efficacy in cancer treatment (13, 14). Moreover, therapies targeting the PD-1 pathway in viral and bacterial infections are emerging (15 – 19). TCR stimulation of CD4^+^ and CD8^+^ T cells induces a transient surface expression of PD-1; however, during cancer and chronic infection, PD-1 cell-surface expression can be imprinted and sustained through antigen-independent stimulation (19 – 21). In relation to both cancer therapy and treatment of chronic viral infections, response to anti-PD-1 therapy is associated with increased activation of CD8^+^ T cells (15, 22).

Engagement of PD-1 by its ligands, PD Ligand-1 or -2 (PD-L1 or PD-L2), inhibits effector T-cell activation, cytokine secretion, and proliferation, but the effect varies according to the strength of the interaction (23). PD-1 ligation leads to the phosphorylation of immunoreceptor tyrosine-based inhibition motif and immunoreceptor tyrosine-based switch motif in the cytoplasmic tail of PD-1, thus recruiting the tyrosine-phosphatase SHP-2, which in complex with PD-1 dephosphorylates intracellular motifs of CD28 and TCR, ultimately attenuating TCR stimulated Ca^2+^ signaling and T cell activation (24, 25).

*S. aureus* has a unique ability to circumvent selective pressures through acquired mutations (26 – 29). We previously showed the combined selective pressure from daptomycin and the host immune system selected for both monocytic natural killer group 2 D (NKG2D)-mediated immune evasion and reduced daptomycin susceptibility through a mutation in the *clpP* gene resulting in the inactivation of the highly conserved ClpP protease (4). Emerging evidence indicates the modulation of PD-1 pathway by several bacterial species, including *Helicobacter pylori, Mycobacterium tuberculosis* (*M. tuberculosis*), *Listeria monocytogenes* (*L. monocytogenes*), and *Escherichia coli* (30 – 33). Few studies have addressed the PD-1 pathway in response to *S. aureus*. Wang et al. reported increased PD-L1 expression on human primary monocytes after *S. aureus* encounter (34), while T_reg_ differentiation was shown to depend on PD-L1 expression on B cells after *S. aureus* exposure (35). Moreover, lymphocytes with increased expression of PD-1 and PD-L1 were shown in furunculosis patients (36), collectively suggesting the involvement of the PD-1 pathway in the anti-staphylococcal immune response. Here we demonstrate that the clinical isolates of *S. aureus* can directly engage with PD-1 to suppress T-cell activation and show that the inhibition can be alleviated by antibody-based blockade of PD-1.

## RESULTS

### *S. aureus* activates human lymphocytes and induces PD-1 surface expression

To examine how T cells respond to clinically derived *S. aureus*, we used a group of five (SADR-1, SADR-2, SADR-3, SADR-4, and SADR-5) methicillin-resistant *S. aureus* (MRSA) strains isolated from the same patient during persistent bacteremia (Fig. S1a). The strains were previously described genetically and phenotypically showing the development of reduced susceptibility to daptomycin as well as resistance to innate NKG2D-ligand-mediated immunity and phagosomal degradation due to a mutation in *clpP* (SADR-2) (4, 26, 27, 29).

**Fig 1:**
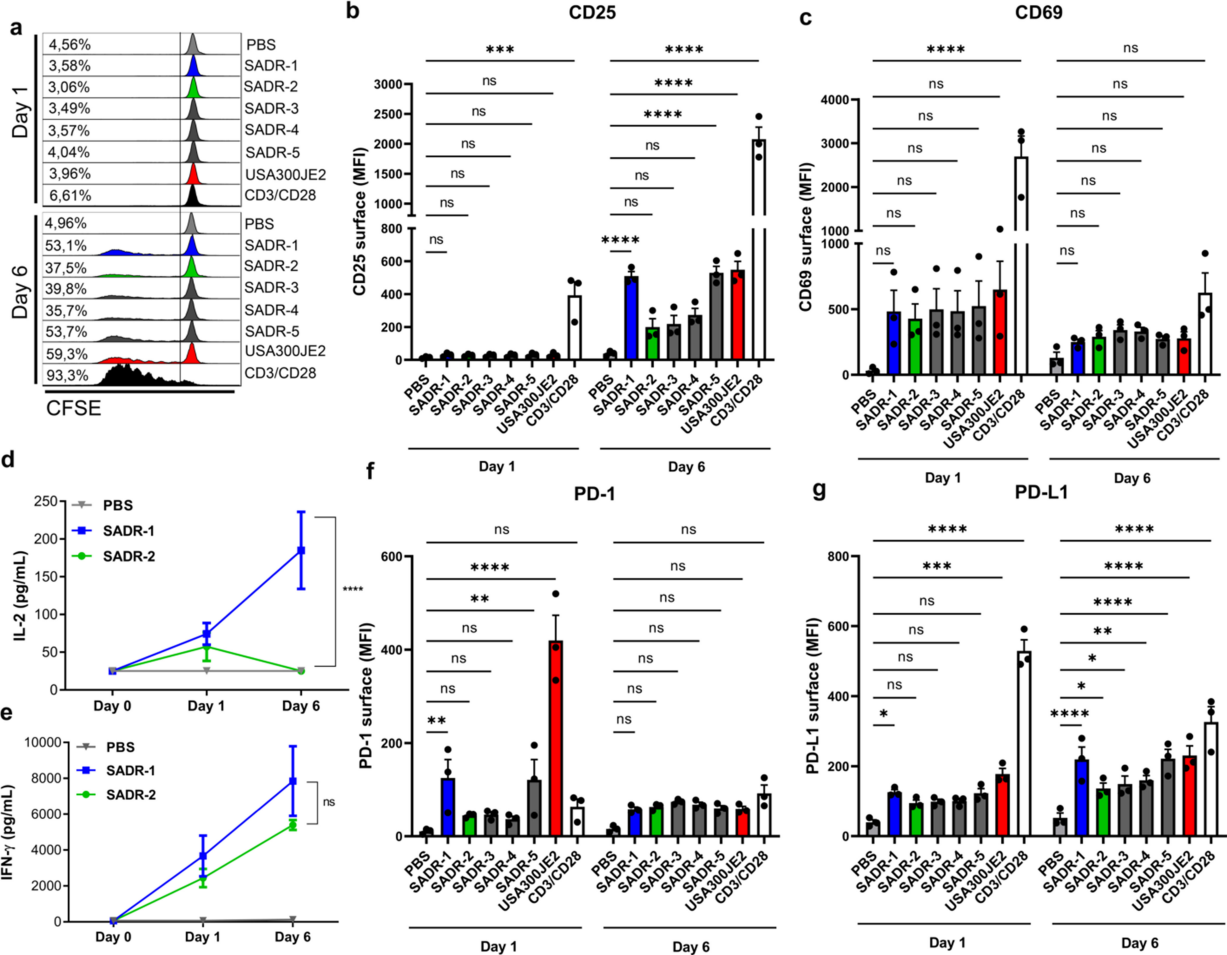
Clinical *S. aureus* activates human T cells. (a–g), Purified human PBLs were treated with PBS, SADR1-5, USA300JE2, or CD3/CD28 beads and analyzed on day 1 and day 6 for proliferation (a), surface CD25 (b), surface CD69 (c), secreted IL-2 (d), secreted IFN-γ (e), surface PD-1 (f), and surface PD-L1 (g). Dotplots and histograms are representative of three independent experiments; results in bargraphs are pooled from three donors (*n* = 3) and presented as mean fluorescence intensity (MFI) or cytokine concentration (pg/mL) ± s.e.m. Statistical analysis was performed by two-way ANOVA with Dunnett’s multiple comparisons test for (b–g). *P < 0.05, **P < 0.01, ***P < 0.001, ****P < 0.0001.

Lymphocyte proliferation in response to UV-inactivated SADR1-5 was examined using carboxyfluorescein succinimidyl ester (CFSE)-labeled peripheral blood lymphocyte (PBL) cultures. All *S. aureus* strains induced proliferation, but there was an obviously reduced response to SADR-2, SADR-3, and SADR-4, compared with SADR-1, SADR-5, and the unrelated MRSA strain USA300JE2 (Fig. 1a; Fig. S1b). As expected, TCR (CD3/CD28) engagement caused a robust response (Fig. 1a; Fig. S1b). In accordance with the proliferation data, only SADR-1, SADR-5, and USA300JE2 significantly induced CD25 surface expression on day 6 after stimulation (Fig. 1b; Fig. S1c), whereas the early T-cell activation marker CD69 was induced to a similar extent by all the strains on day 1 (Fig. 1c), pointing toward a partial abrogation of the late response to the SADR-2, SADR-3, and SADR-4 isolates. There was no significant difference in cell viability in response to treatment with either *S. aureus* strain (Fig. S1d), ruling out that SADR-2-, SADR-3-, and SADR-4-mediated toxicity caused the reduced proliferation and CD25 expression. In accordance with the observed lymphocyte activation, SADR-2, SADR-3, and SADR-4 caused less IL-2 secretion on day 6 (Fig. 1d; Fig. S1e), while all *S. aureus* strains induced IFN-γ secretion to a similar extent (Fig. 1e; Fig. S1f).

Surface expression of PD-1 is tightly linked to T-cell activation (37). Interestingly, we found that PD-1 was significantly induced on the surface of lymphocytes on day 1 after SADR-1, SADR-5, and USA300JE2 exposure, while less PD-1 was detected on cells stimulated with SADR-2, SADR-3, and SADR-4 (Fig. 1f; Fig. S1c). On day 6 after stimulation, the induced PD-1 expression almost returned to normal, and no difference between the strains was detected (Fig. 1f; Fig. S1c). Similarly, PD-L1 was significantly induced on day 1 after SADR-1 and USA300JE2 exposure while all strains significantly induced PD-L1 on day 6 (Fig. 1g). Together, these data indicate that lymphocytes respond to *S. aureus* by inducing proliferation and surface expression of activation markers including PD-1, while mutations that reduce *S. aureus* susceptibility to daptomycin seem to partly abrogate this stimulation.

### *ClpP* mutant *S. aureus* inhibits human lymphocyte activity

**Fig 2:**
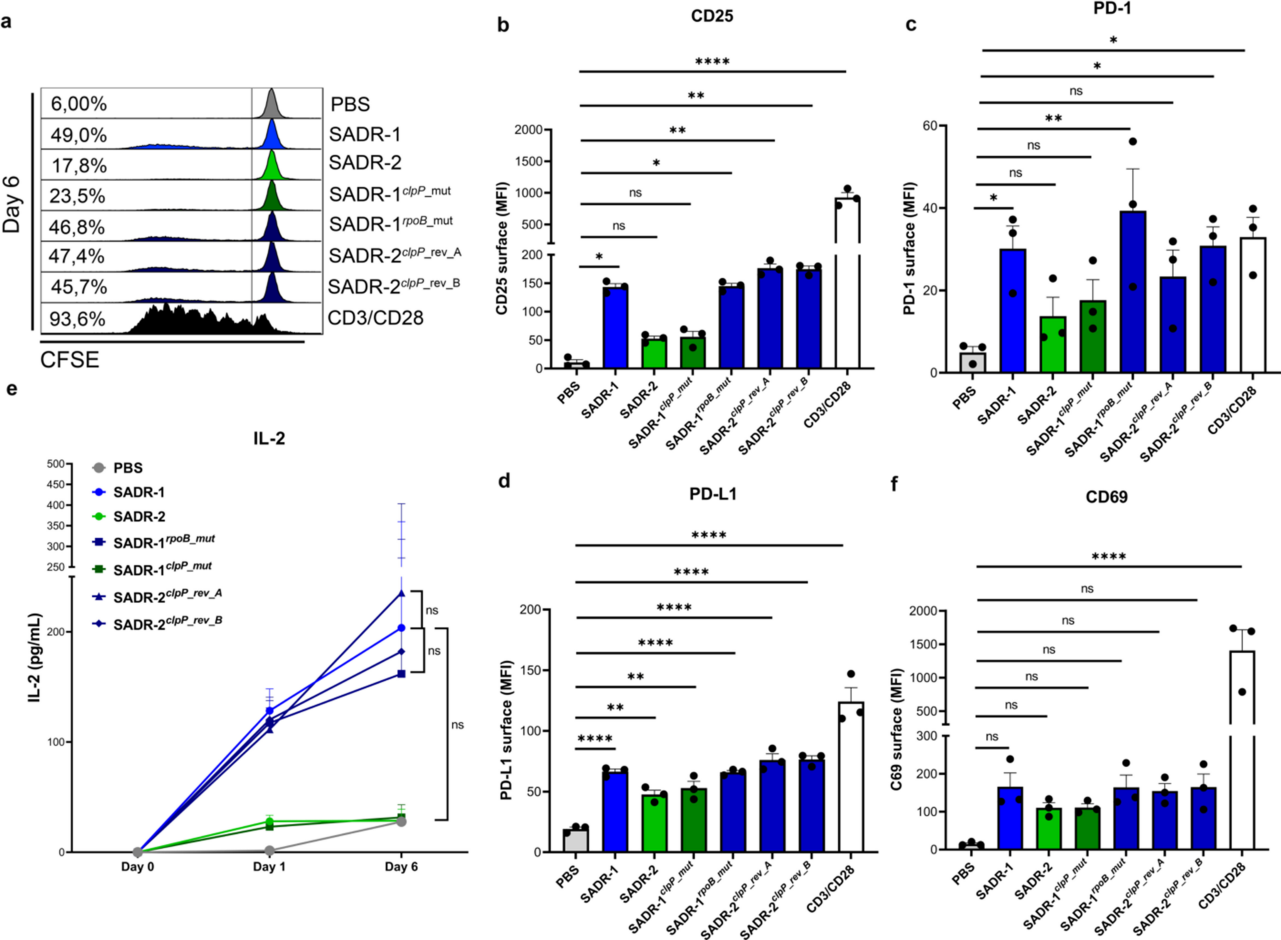
Reversion of *clpP* in SADR-2 rescues the T-cell suppressing phenotype of *S. aureus*. (a–f), Purified human PBLs were treated with PBS, SADR-1, SADR-2, SADR-1*^rpoB_mut^*, SADR-1*^clpP_mut^*, SADR-2*^clpP_rev_A^*, SADR-2*^clpP_rev_B^*, or CD3/CD28 beads and analyzed on day 6 for proliferation (a), surface CD25 on day 6 (b), surface PD-1 on day 1 (c), surface PD-L1 on day 6 (d), secreted IL-2 on day 1 and day 6 (e), surface CD69 on day 1, statistics show SADR-2 comparison with SADR-1, SADR-1 comparison with SADR-1*^rpoB_mut^*, and SADR-1 comparison with SADR-2*^clpP_rev_A^* (f). Histograms are representative of three independent experiments; results in bargraphs are pooled from three donors (*n* = 3) and presented as mean fluorescence intensity (MFI) or cytokine concentration (pg/mL) ± s.e.m. Statistical analysis was performed by one-way ANOVA with Dunnett’s multiple comparisons test in panels b–d and f and two-way ANOVA with Tukey’s multiple comparisons test in panel e. *P < 0.05, **P < 0.01, ****P < 0.0001.

**Fig 3:**
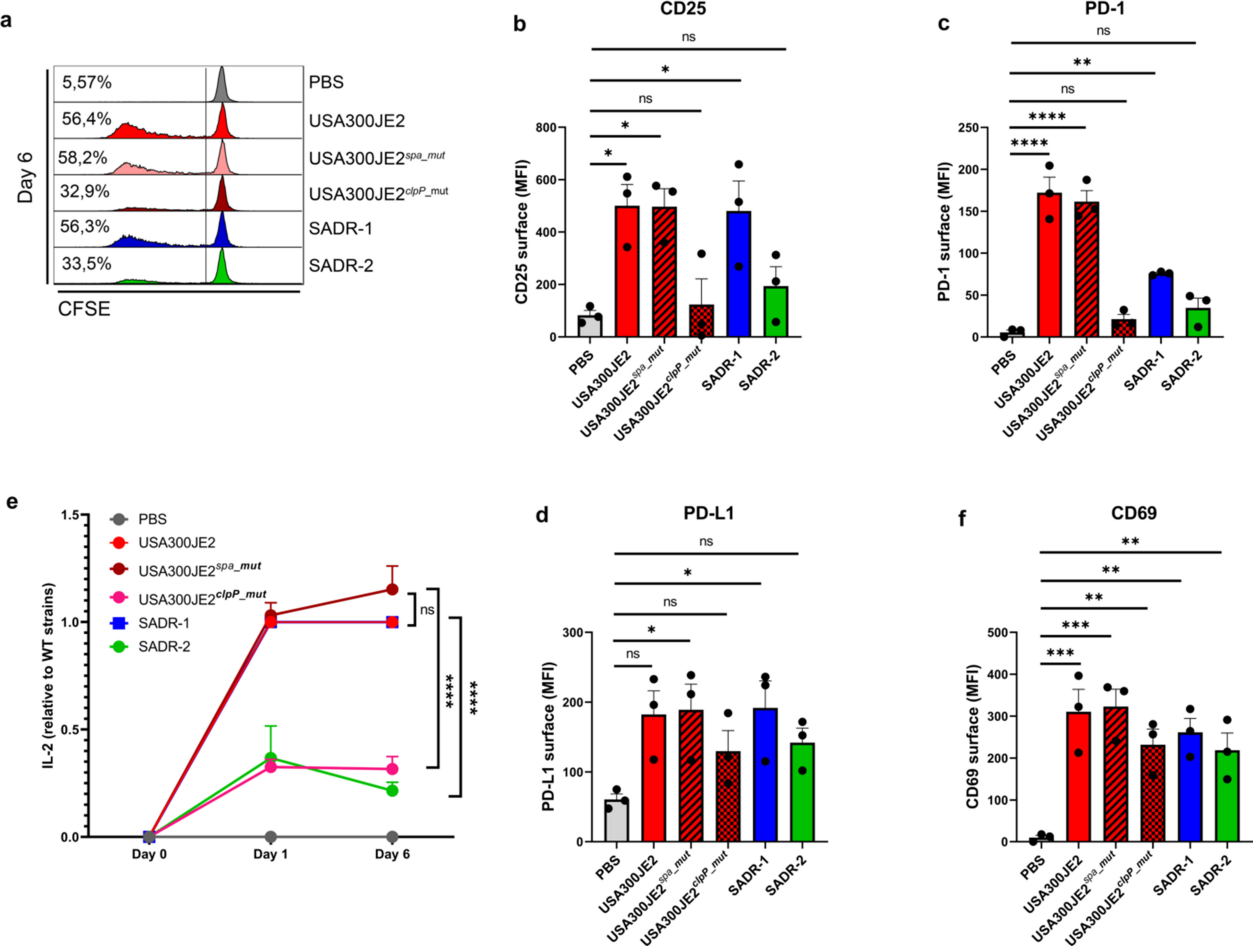
Mutation of *clpP* in USA300JE2 promotes the T-cell suppressing phenotype of *S. aureus*. (a–f), Purified human PBLs were treated with PBS, USA300JE2, USA300JE2*^spa_mut^*, USA300JE2*^clpP_mut^*, SADR-1, or SADR-2 and analyzed on day 6 for proliferation (a), surface CD25 on day 6 (b), surface PD-1 on day 1 (c), surface PD-L1 on day 6 (d), secreted IL-2 on day 1 and day 6 relative to wild-type strains (USA300JE2 and SADR-1, respectively) (e), surface CD69 on day 1 (f). Histograms are representative of three independent experiments; results in bargraphs are pooled from three donors (*n* = 3) and presented as mean fluorescence intensity (MFI) or cytokine concentration (pg/mL) ± s.e.m. Statistical analysis was performed by one-way ANOVA with Dunnett’s multiple comparisons test in panels b–d and f and two-way ANOVA with Tukey’s multiple comparisons test in panel e.*P < 0.05, **P < 0.01, ***P < 0.001, ****P < 0.0001.

To examine how SADR-2 inhibits lymphocyte activation, we compared the specific differences between the two isogenic strains SADR-1 and SADR-2. SADR-2 carries single-nucleotide polymorphisms (SNPs) in the *clpP* and *rpoB* genes (Fig. S1a), and to determine the contribution of the individual mutations, we introduced each SNP separately into the parental SADR-1 strain. Introduction of the *rpoB* mutation (SADR-1*^rpoP^* ^_mut^) had no effect on lymphocyte proliferation (Fig. 2a; Fig. S2a) or induced expression of CD25 (Fig. 2b) and PD-1 (Fig. 2c). In contrast, introducing the *clpP* mutation in SADR-1 (SADR-1*^clpP^* ^_mut^) phenocopied SADR-2, and similarly reverting the mutant *clpP* allele in SADR-2 back to wild-type (SADR-2*^clpP^* ^_rev_A^ and SADR-2*^clpP^* ^_rev_B^) phenocopied SADR-1 with respect to proliferation (Fig. 2a), CD25 (Fig. 2b), PD-1 (Fig. 2c), PD-L1 (Fig. 2d), and IL-2 secretion (Fig. 2e). As expected, CD69 surface expression was not significantly affected by either mutation or reversion (Fig. 2f). Together, these data clearly demonstrate that the single-nucleotide change in *clpP* is responsible for the reduced activation of lymphocytes observed for SADR-2. The mutation in *clpP* changes a highly conserved glycine residue (G94), which is located in close proximity to the active site serine (S_98_) (38), indicating that this SNP may inactivate the function of ClpP. In support here off, a loss-of-function mutation in *clpP* in the USA300JE2 (USA300JE2*^clpP^* ^_mut^) reduced lymphocyte activation to a similar extent as the *clpP* mutation in SADR-1, while introducing an unrelated mutation in Protein A (USA300JE2*^spa^* ^_mut^) did not affect lymphocyte activity (Fig. 3a through f; Fig. S2c). The specific impact of ClpP in SADR-2 was further established by testing four clinically relevant *rpoB* mutations in a USA300 (clone NRS384) *S. aureus* strain (28, 39, 40) (Fig. S2e through S2i). No change in cell viability was observed in response to either strain background (Fig. S2b, S2d and S2j). These results emphasize the importance of ClpP activity in *S. aureus* for the activation of human lymphocytes.

### *S. aureus* directly mobilizes Ca^2+^ flux in human T cells and induces PD-1

**Fig 4:**
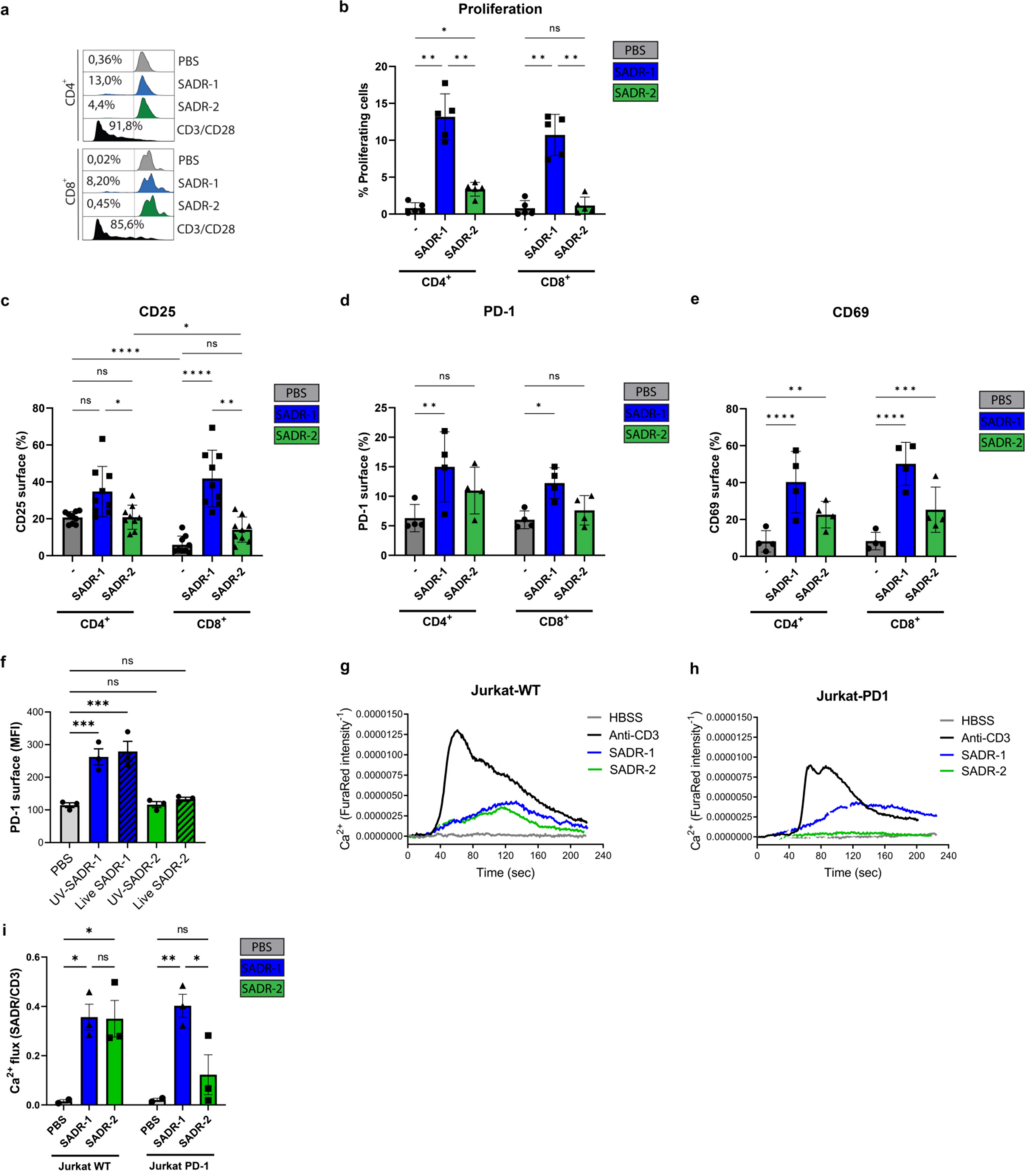
*S. aureus* directly activates purified T cells and intracellular Ca2^+^ flux. (a–e), Purified human CD3^+^ T cells were treated with PBS, SADR-1, SADR-2, or CD3/CD28 and analyzed on day 5 for proliferation (a–b), surface expression of CD25 (c), PD-1 (d), and CD69 (e). (f), Jurkat Tag9 cells were stimulated with UV-killed or live SADR-1, SADR-2 or PBS and analyzed on day 1 for surface expression of PD-1. (g–i), Jurkat T cells were loaded with Fura2Red for Ca^2+^ flux analysis. Cells were stimulated with anti-CD3 (1 µg/mL), SADR-1, SADR-2, or HBSS (untreated control). Representative Ca^2+^ responses are shown as ratio between the fluorescence intensities from excitation at 340 and 380 nm for Jurkat-WT (g) and Jurkat-PD1 (h), and collected data from three independent experiments (*n* = 3) are presented (i). The data are shown as representative data from one donor in panel a, mean ± SD from independent experiments, presenting % surface expression on CD8^+^ or CD4^+^ T cells (*n* = 5) in panel b, (*n* = 8) in panel c, and (*n* = 4) in panels d and e, mean fluorescence intensity (MFI) (*n* = 3) in panel f, and Ca^2+^ flux relative to anti-CD3 stimulation (*n* = 3) in panel i. Statistical analysis was performed by two-way ANOVA with Sidak’s multiple comparisons test in panels b–e, one-way ANOVA with Dunnett’s multiple comparisons test in panel f, and two-way ANOVA with Tukey’s multiple comparisons test in panel i.*P < 0.05, **P < 0.01, ***P < 0.001, ****P < 0.0001.

Since PBL cultures not only consist primarily of T cells but also contain other lymphocytes, including B and NK cells, we next sought to examine T-cell activation by *S. aureus* using purified CD3^+^ T cells (Fig. S3a through S3b). Purified T cells behaved like PBL cultures upon *S. aureus* exposure by inducing substantially higher proliferation (Fig. 4a and b) as well as expression of CD25 (Fig. 4c), PD-1 (Fig. 4d), and CD69 (Fig. 4e) in response to SADR-1 compared with SADR-2, thus confirming a direct activation of T cells. Of note, the induction of CD69 was more pronounced in pure CD3 cultures (Fig. 4e) compared with PBLs (Fig. 1c) indicating differences in the kinetics of activation between the culture conditions. Notably, proliferation and CD25 expression were higher in response to SADR-1 compared with SADR-2 for both CD4^+^ and CD8^+^ T cells (Fig. 4a through c). However, the difference between SADR-1 and SADR-2 induced stimulation appeared more pronounced for CD8^+^ T cells, and while CD4^+^ T cells proliferated in response to both SADR-1 and SADR-2, there was a specific lack of proliferative response to SADR-2 from the CD8^+^ T cells (Fig. 4a and b).

To further confirm the direct activation of T cells by *S. aureus,* we next turned to CD4^+^ Jurkat T cells that induced PD-1 surface expression (Fig. S3c) similar to PBLs (Fig. S1c) after exposure to *S. aureus*. We further confirmed that both live and UV-killed *S. aureus* induced similar expression of PD-1 on Jurkat T cells (Fig. 4f). Intracellular Ca^2+^ flux is a vital early step in the signaling cascade of activated T cells (41, 42). Compared with anti-CD3, which initiated a classical abrupt increase in free intracellular Ca^2+^, both SADR-1 and SADR-2 induced a similar but more protracted Ca^2+^ flux in Jurkat WT T cells (Jurkat-WT) (Fig. 4g through i). To examine the importance of PD-1 expression, we used Jurkat T cells with stable overexpression of PD-1 on the surface (Jurkat-PD1) (24). Stimulation with anti-CD3 or SADR-1 induced Ca^2+^ responses similar to Jurkat-WT cells, but strikingly no Ca^2+^ mobilization was detected in SADR-2-exposed Jurkat-PD1 cells (Fig. 4h and i). These data thus suggest that both SADR-1 and SADR-2 directly activate and induce a Ca^2+^ flux in naïve PD-1^low^ T cells; however, PD-1 surface expression inhibits the Ca^2+^ mobilization in response to SADR-2 exposure.

### *S. aureus* with *clpP* mutation interacts directly with PD-1 expressed on T cells

**Fig 5:**
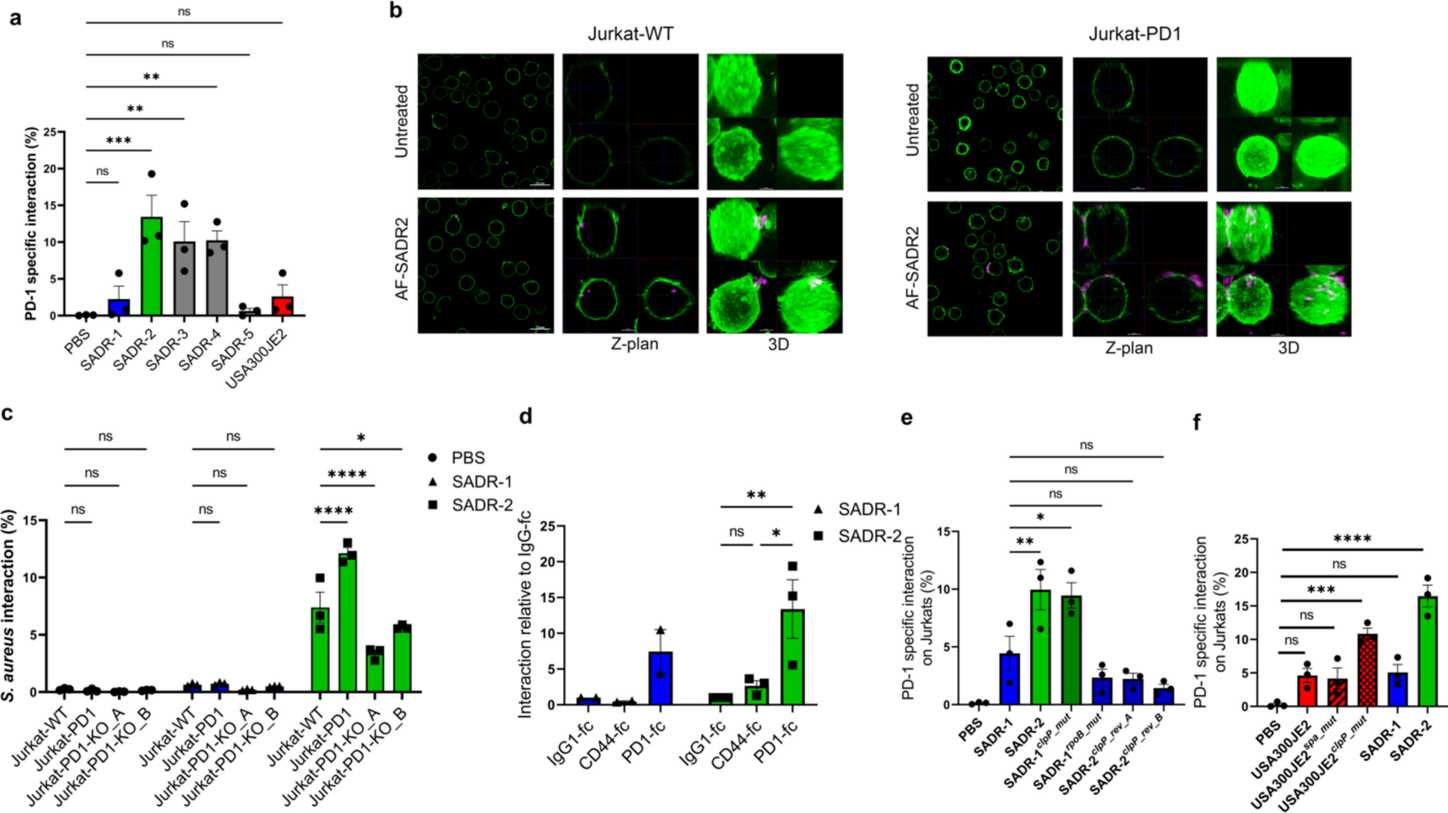
*ClpP* mutant *S. aureus* interacts directly with PD-1. (a–f), Jurkat-PD1 and Jurkat-WT cells were treated with PBS or AF647-labeled *S. aureus* strains. PD-1-specific interaction was analyzed after 14–20 h by flow cytometry and presented as % *S. aureus* interaction with Jurkat-PD1 cells subtracted interaction with Jurkat-WT cells. (a) Confocal microscopy: cell surface (green) and *S. aureus* (magenta). Overview of cells (left row; bar, 20 µm), selected Z-plan projected as X- vs Y-axis (middle row; bar, 5 µm), and 3D structure presented as maximal intensity projection (right row; bar, 5 µm) (b). Jurkat-PD1, Jurkat-WT, and Jurkat-PD1-KO T cells were treated with PBS- or AF647-labeled SADR-1 or SADR-2. Interaction was analyzed after 2 h by flow cytometry (c). Interaction of SADR-1 and SADR-2 with soluble chimeric PD-1-fc, CD44-fc, and IgG-fc by flow cytometry. Data show the percentage interaction relative to IgG-fc control (*n* = 3) for SADR-2 and (*n* = 2) for SADR-1 interaction (d). Jurkat-PD1 and Jurkat-WT cells were treated with PBS- or AF647-labeled *S. aureus* strains. PD-1-specific interaction was analyzed after 14–20 h by flow cytometry for SADR-1, SADR-2, SADR-1^rpoB_mut^, SADR-1^clpP_mut^, SADR-2^clpP_rev_A^, and SADR-2^clpP_rev_B^ (e) and USA300JE2, USA300JE2^spa_mut^, USA300JE2^clpP_mut^, SADR-1, or SADR-2 (f). The data are shown as mean ± s.e.m. Collected data (*n* = 3) are presented as % interaction in panels a, c, e, and f) and as interaction relative to IgG1-fc in panel d. Statistical analysis was performed by one-way ANOVA with Dunnett’s multiple comparisons test in panels a, e, and f); two-way ANOVA with Dunnett´s multiple comparisons test in panel c; and two-way ANOVA with Tukey’s multiple comparisons test in panel d).*P < 0.05, **P < 0.01, ***P < 0.001, ****P < 0.0001.

Exhausted and unresponsive tumor-infiltrating T cells often express high levels of PD-1, and intracellular Ca^2+^ flux is one of the early T-cell responses that is most sensitive to PD-1 inhibition (23, 43, 44). The abrogated Ca^2+^ mobilization in PD-1 overexpressing T cells mediated by SADR-2 (Fig. 4g through i) suggests that PD-1 could be directly involved in T-cell inhibition after SADR-2 exposure. We therefore examined if SADR-2 directly interacts with PD-1 on the surface of the T cells. Using fluorescently labeled *S. aureus* (4), we found that SADR-2, SADR-3, and SADR-4 interacted significantly more with Jurkat-PD1 compared with Jurkat-WT, while SADR-1, SADR-5, and USA300JE2 showed significantly lower PD-1-specific interaction (Fig. 5a). SADR-2 interaction with Jurkat-PD1 was validated by confocal microscopy (Fig. 5b), and knockout (KO) of PD-1 largely reduced the interaction of SADR-2 with Jurkat T cells further confirming that PD-1 is essential for binding to SADR-2 (Fig. 5c). PD-1 overexpression and KO were confirmed by flow cytometry (Fig. S4a). We examined the specificity of the interaction between PD-1 and *S. aureus* using a cell-free system assessing the interaction between *S. aureus* and soluble recombinant PD-1-Fc chimeric receptor. Indeed, the *clpP* mutant SADR-2 showed significantly increased binding to PD-1-Fc compared with IgG-fc, whereas substantially lower interaction was observed for PD-1-Fc and SADR-1 (Fig. 5d). As expected (45), no interaction was observed with the control chimeric proteins CD44-Fc (Fig. 5d). These data strongly suggest that the inhibited phenotype of SADR-2-exposed T cells is caused by direct inhibitory interaction with PD-1.

By examining the involvement of *clpP* and *rpoB* in PD-1 interaction, we found that the mutation in *rpoB* in SADR-1 (SADR-1*^rpoP^* ^_mut^) did not affect the PD-1-specific interaction, whereas the introduction of the *clpP* mutation (SADR-1*^clpP^* ^_mut^) significantly increased the PD-1 interaction (Fig. 5e). Likewise, reverting *clpP* in SADR-2 (SADR-2*^clpP^* ^_rev_A^ and SADR-2*^clpP^* ^_rev_B^) significantly inhibited the PD-1 interaction (Fig. 5e). Moreover, the less closely related USA300JE2*^clpP^* ^_mut^ strain also interacted with PD-1 on Jurkat T cells (Fig. 5f), and finally, the *rpoB* substitution A477D present in SADR-2 did not alter PD-1 interaction in the NRS384-USA300 *S. aureus* background (Fig. S4b). *S. aureus* labeling was confirmed by flow cytometry (Fig. S4c through S4e). In conclusion, these data suggest that *clpP* mutant *S. aureus* induces a PD-1-binding molecule on its surface that inhibits T cells through PD-1 engagement.

### PD-1 blockade alleviate *clpP* mutant SADR-2-mediated T cell inhibition

**Fig 6:**
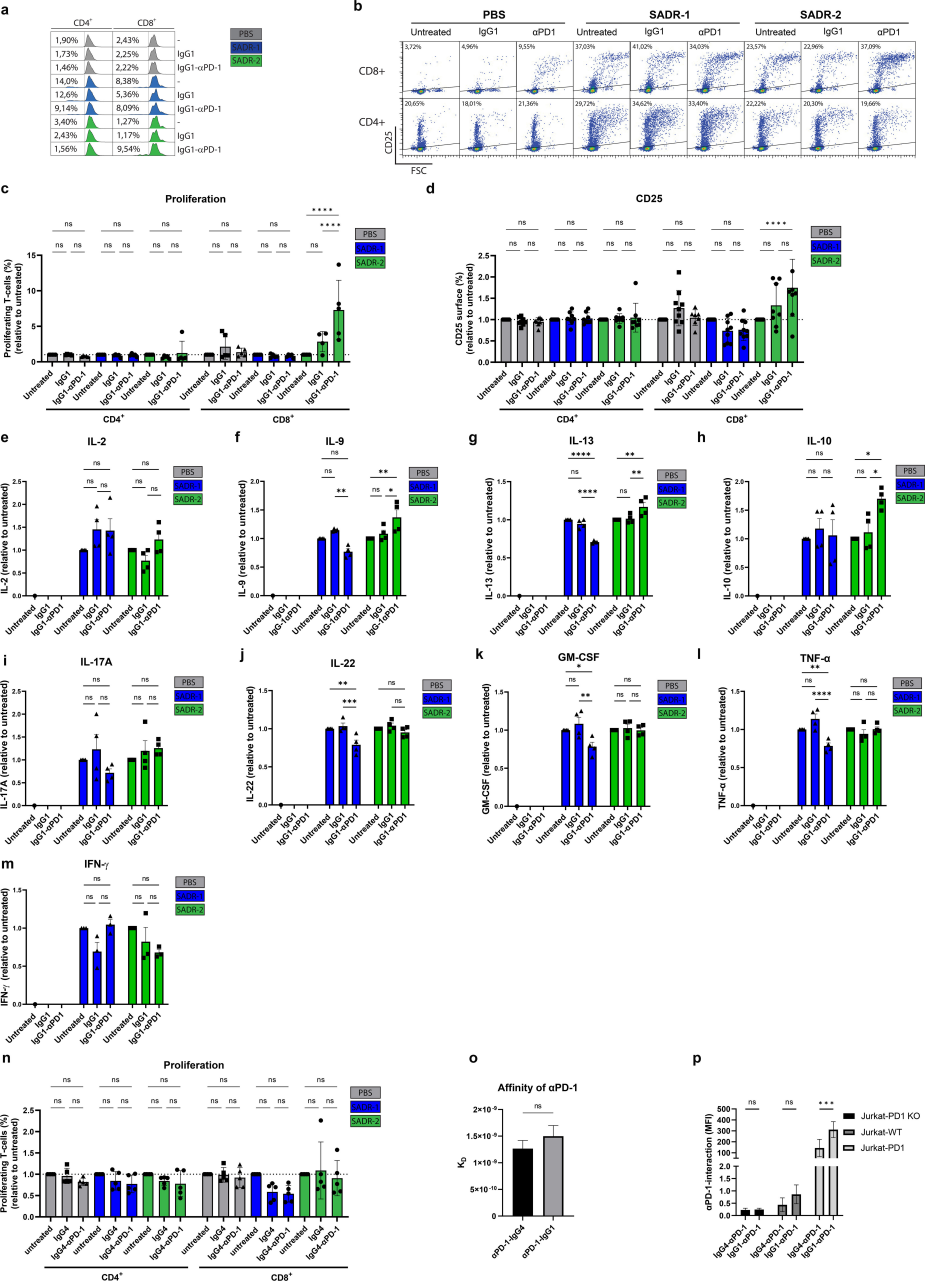
PD-1 blockade rescues T-cell function after SADR-2 exposure. (a–m), Purified human CD3^+^ T cells were treated with PBS, SADR-1, or SADR-2 alone or combined with IgG1- or IgG4-based anti-PD-1 (IgG1-αPD1 and IgG4-αPD1, respectively) and analyzed on day 5 for proliferation (a, c, and n), surface expression of CD25 (b+d), and secreted IL-2 (e), IL-9 (f), IL-13 (g), IL-10 (h), IL-17A (i), IL-22 (j), GM-CSF (k), TNF-α (l), and IFN-γ (m). Proliferation histograms are representative of five independent experiments, while CD25 dotplots are representative of eight independent experiments. Collected data are presented as mean +/− SD of % surface expression in panel d (*n* = 8) and mean +/− s.e.m. of pg/mL relative to untreated samples in panels e–m (*n* = 4). The affinity of IgG1- and IgG4-αPD1 to recombinant PD-1 was compared using biolayer interferometry (o). The bar graph shows mean +/− SD of K_D_ values determined in three independent experiments for each antibody. Specificity and strength of the interaction of IgG1- and IgG4-αPD1 with cell-displayed PD-1 were investigated by incubating labeled antibodies with Jurkat PD-1 KO, Jurkat-WT, and Jurkat-PD1 cells for 10 min on ice followed by analysis using flow cytometry (p). Collected data from three independent experiments using an antibody concentration of 6 ug/mL are presented as the mean +/− SD of the geometric mean fluorescent intensity (MFI). Statistical analysis was performed by two-way ANOVA with Tukey’s multiple comparisons test in panels c–n and p and by an unpaired *t*-test in panel o. *P < 0.05, **P < 0.01, ***P < 0.001, ****P < 0.0001.

To test whether the interaction of SADR-2 with PD-1 is responsible for the inhibited T-cell phenotype, we co-cultured the T cells with an engineered IgG1-based PD-1 blocking antibody (IgG1-αPD1) based on the variable domains of the therapeutically available IgG4-based Nivolumab (IgG4-αPD1). Treatment of T cells with IgG1-αPD1 in combination with SADR-2 increased proliferation and CD25 expression on CD8^+^ T cells, whereas no response was observed by IgG1-αPD1 alone or combined with SADR-1 (Fig. 6a through d). CD69 expression was not affected by blocking PD-1 (Fig. S5a and S5b). PD-1 blockade was confirmed by flow cytometry (Fig. S5c and S5d). Interestingly, the observed effect of IgG1-αPD1 was only observed for CD8^+^ T cells (Fig. 6a through d) and only when stimulated in cultures also containing CD4^+^ T cells, thus not in pure CD8^+^ T cells (Fig. S5e).

To assess the impact on cytokine responses, we analyzed supernatants from PD-1-blocked T cells (Fig. 6e through m; Fig. S5g through S5p). We found a strong trend that PD-1 blockade increased IL-2 production from SADR-2-exposed T cells (Fig. 6e; Fig. S5g). Interestingly, IL-9, IL-13, and IL-10 were significantly increased in SADR-2-exposed T cells (Fig. 6f through h). Moreover, PD-1 blocking of SADR-2-exposed T cells increased IL-17A in three out of four donors (Fig. 6i), while no change was observed for IL-22 (Fig. 6j), GM-CSF (Fig. 6k), TNF-α (Fig. 6i), and IFN-γ (Fig. 6m). In SADR-1-exposed T cells, several cytokines were down-modulated by blocking PD-1, including IL-9 (Fig. 6f), IL-22 (Fig. 6j), IL-17A (Fig. 6i), GM-CSF (Fig. 6k), TNF-α (Fig. 6i), and IL-13 (Fig. 6g). Finally, in line with the dependency of mixed CD4^+^ and CD8^+^ T-cell cultures observed for CD25 rescue (Fig. 6d; Fig. S5e), no cytokines were increased after PD-1 block and SADR-2 exposure in pure CD8^+^ T-cell cultures (Fig. S6a through S6g). To focus on the direct effect of the αPD1 antibody, all data are normalized to untreated, SADR-1, or SADR-2 treatment/exposure, respectively (Fig. 6), and non-normalized data are presented in Fig. S5 and S6.

In contrast to the engineered IgG1-αPD1, the therapeutically available IgG4-based PD-1-blocking antibodies Nivolumab (IgG4-αPD1) (Fig. 6n) and Pembrolizumab (data not shown) had no effect on *S. aureus*-induced T-cell proliferation (Fig. 6n) or CD25 (Fig. S5e) expression. To investigate the mechanism behind the different blocking efficiency of IgG1- and IgG4-based αPD1, we compared their affinities to recombinant PD-1 using biolayer interferometry (BLI) (Fig. 6o) as well as their interaction with PD-1 displayed on Jurkat T cells (Fig. 6p). While the antibodies showed comparable affinities to recombinant soluble PD-1, IgG1-αPD1 interacted significantly more with PD-1 displayed on Jurkat T cells (Jurkat-PD1 and Jurkat-WT).

In conclusion, effective PD-1 blocking by IgG1-αPD1 restored several inhibited effector functions in *clpP* mutant SADR-2-exposed T cells, while these effects were not observed in SADR-1-exposed T cells.

## DISCUSSION

T cells are fundamental for the successful elimination of bacterial infections and development of immunological memory (46). To assess our hypothesis that *S. aureus* infection in humans involves direct staphylococcal-mediated adaptive immune evasion, we investigated T-cell responses to clinically derived *S. aureus* isolates that developed under selective pressure from the host immune system and daptomycin treatment (26, 27). So far, *S. aureus* mutations have mainly been characterized for their contribution to antibiotic resistance (47), while less is known about how these recurrent mutations are linked to immune evasion. We find that a clinically selected *S. aureus clpP* mutant directly inhibits T-cell function through interaction with PD-1 on activated T cells. ClpP is, along with ClpX, part of the staphylococcal proteolytic system, which is essential for regulating virulence, antibiotic resistance, immune evasion, and adaptation to the changing environment in the host (38, 48 – 50). Interestingly, therapeutics targeting ClpP have emerged (51), and *S. aureus* with *clpP* mutations is advancing with increasing incidences of vancomycin and daptomycin non-susceptible MRSA strains (4, 27, 29, 52 – 54). We have previously shown that isolates loosing ClpP function fail to activate human monocytes, thus likely gaining immune evasive advantages (4). Furthermore, we found that SADR-3 and SADR-4 (with mutation in *clpX*) inhibited T-cell proliferation, CD25 expression, and IL-2 secretion to a similar extent as *clpP* mutant *S. aureus* (SADR-2). We speculate that the combined effect of ClpP-ClpX in *S. aureus* drives the PD-1 interaction and subsequent T-cell repression. Despite the essential role of ClpP for *S. aureus* virulence in animal models of infection (48, 55 – 57), mutations in the *clpP* gene on multiple occasions have been identified in *S. aureus* strains isolated from patients undergoing treatment with daptomycin or vancomycin, raising the question of how *S. aureus* benefits from mutations in the ClpP protease in patients treated with these last-line antibiotics. The current data, as well as our previous findings, indicate that related immune evasive mechanisms could explain the selection of *clpP* mutations in the host.

Antibiotic treatment and host immunity alter *S. aureus* expression of both secreted and surface-bound virulence factors (58). Our work has focused on the response to staphylococcal surface structures, which thus excludes possible confounding interactions from secreted factors including superantigens (12), although PD-1 induction was observed also by live *S. aureus*. We found that *S. aureus* directly activated human T cells, and this activation increased the surface expression of PD-1, which, in contrast to TCR stimulation, was not associated with increased PD-1 mRNA levels (Fig. S7a and b), supporting the notion that *S. aureus* activates T cells through a new and undescribed pathway independent of TCR and superantigens. Moreover, we found that the difference in the activation of T cells responding to SADR-1 and SADR-2 was observed for CD25 but not for CD69, which we ascribe to the difference in the timing of expression of the two activation markers. Future work should investigate the interacting molecules on *S. aureus* and human T cells to understand how T cells are activated by *S. aureus* and how this leads to PD-1 regulation and interaction.

Interestingly, blocking of PD-1 with an engineered IgG1-based αPD-1-specific antibody based on Nivolumab variable domains abrogated the inhibitory effect of SADR-2 on proliferation and CD25 expression on CD8^+^ T cells and IL-2 secretion. This finding corresponds well to the specific lack of proliferative response to SADR-2 stimulation in CD8^+^ T cells (Fig. 4b) as well as the correlation between increased CD8^+^ T-cell activation and response to anti-PD-1 therapy in patients with cancer and chronic virus infections (15, 22). In contrast to αPD-1 IgG1, clinically available IgG4-based αPD-1 antibodies (Pembrolizumab and Nivolumab) did not restore T-cell activation upon *clpP* mutant *S. aureus* exposure. The functional differences between IgG1- and IgG4-based PD-1 blockade correlate with an increased binding of IgG1- versus IgG4-based αPD-1 with PD-1 displayed on Jurkat cells (Fig. 6p), while BLI confirmed that the two antibodies, containing identical variable domains, have comparable affinities toward recombinant human PD-1 (Fig. 6o). While we cannot completely rule out that the apparent difference in binding of IgG1- and IgG4-based αPD-1 to Jurkat cells is partially caused by differences in detection rather than true differences in interaction with cell-displayed PD-1, the combined data suggest that isotype of the antibody plays a significant role for their blocking efficacy, potentially via differences in the flexibility of the hinge regions which will be subject for further investigations. Purified CD8^+^ T cells were directly activated by *S. aureus*; however, PD-1 antibody rescue of activation in SADR-2-exposed CD8^+^ T cells depended on the presence of CD4^+^ T cells, which suggest a complex interaction between T-cell subtypes, potentially involving cytokines, that clearly warrants further investigation.

Our findings suggest that blocking *S. aureus*-mediated PD-1 inhibition of T cells can potentially be used therapeutically to reinvigorate immunity toward specific *S. aureus* isolates. In cancer treatment, variations in patient responses are well described, although not fully understood (13, 59). Likewise, we observed donor variation in the restoration of T-cell activation after PD-1 blockade of SADR-2-stimulated cells, and we speculate whether this could be influenced by differences in *S. aureus* carrier status since previous studies have reported that staphylococcal immunity is greatly influenced by *S. aureus* carrier status (60, 61). Moreover, we found that T cells from non-carriers showed higher induction of surface activation markers, in particular PD-1 in response to SADR-1 and USA300JE2 with no differences in toxicity (Fig. S7c through e).

*L. monocytogenes* infection in mice showed an increase in PD-1 and PD-L1 on both CD4^+^ and CD8^+^ T cells (62, 63). However, these studies found that blocking the PD-1 pathway resulted in failure to clear infection. Correspondingly, PD-1 KO mice showed significantly reduced survival in response to *M. tuberculosis* (64). These studies thus suggest a protective effect of PD-1 signaling with these pathogens; it is, however, unclear how this translates to human infection with *L. monocytogenes* or *M. tuberculosis*. The use of mouse models for *in vivo* studies on *S. aureus* is debated since mice are not natural staphylococcal hosts (65, 66). In addition, SADR-2 is selected for binding to human PD-1, and therefore, we do not expect SADR-2 to interact equally with murine PD-1, which challenges the use of mouse models for *in vivo* testing. Our data using clinically derived *S. aureus* isolates and PD-1 overexpression or KO and purified recombinant PD-1 clearly show that *clpP* mutant *S. aureus* directly interacts with PD-1.

Pathogens targeting the PD-1 system usually affect the endogenous expression of PD-1 receptor or its ligands (15, 18, 30, 31). We show that certain (*clpP*/*X* mutant) *S. aureus* expresses a molecule/structure that directly binds and targets PD-1 signaling that is likely highly advantageous for the bacteria as the inhibitory effect occurs without further involvement of the host. However, this likely results in strong selective pressure of the host to neutralize the PD-1 interaction, possibly by antibodies. We speculate that *S. aureus* has formed a niche to advance survival in which the PD-1 interacting molecule is only induced after a potent host immune attack, which agrees with the SADR-2, SADR-3, and SADR-4 phenotypes formed by *in vivo* selection pressure. Moreover, the affinity for the PD-1 interaction is likely carefully selected, as a strong interaction could result in down-modulation or blocking of PD-1, which would not be beneficial for *S. aureus*.

In summary, *S. aureus* directly activates human T cells and induces PD-1 surface expression. In addition, we describe a novel immune evasive mechanism whereby *clpP* mutant *S. aureus* inhibits Ca^2+^ flux, CD25 expression, and IL-2 secretion via interaction with PD-1; and we show that PD-1 blockade can restore several T-cell functions. Collectively, we identify the PD-1 pathway as a novel immune evasive mechanism used by *S. aureus* to manipulate T-cell immunity. Given the successful use of therapeutic antibodies blocking PD-1 in cancer, we see a significant potential for a similar use in the treatment of certain persistent staphylococcal infections.

## MATERIALS AND METHODS

### Human cells and culture conditions

Jurkat Tag9 (JTag9) T cells were kindly provided by Dr. Carsten Geisler (Department of Immunology and Microbiology, University of Copenhagen, Denmark). Jurkat E6-1 (Jurkat-WT) T cells and Jurkat E6-1 cells stably overexpressing PD1 (Jurkat-PD1) cells were a kind gift from Dr. Enfu Hui (Division of Biological Sciences, University of California, San Diego, United States) (24). Jurkat PD1 KO cells were created using the CRISPR/Cas9 system described previously (67). Jurkat-WT cells were maintained in RPMI 1640 (Gibco, 32404014) supplemented with 10% FBS (Gibco, 11550356), 2 mM L-glutamine (Sigma, G7513) and penicillin/streptomycin (100×, Gibco, 15140122) at 37°C, and 5% CO_2_. Cells were seeded at a density of 2.5 × 10^5^ cells/mL one day prior to transfection. Electroporation was conducted with 1 × 10^6^ cells and 1  µg each of endotoxin-free plasmid DNA of CAS9PBKS (Addgene Plasmid, 68371) and gRNA in the plasmid U6GRNA (Addgene Plasmid, 68370) using 4D-Nucleofector Kit SE and program CK-116 with Lonza 4D-Nucleofector System (Lonza, Switzerland). FACS enrichment of GFP-positive cells was conducted 2 days after transfection using flow cytometry-based cell sorting with Sony MA900 (Sony Biotechnologies, Japan). These enriched cells were cultured for more than two weeks before single-cell sorting into 96-well plates. KO clones were screened by flow cytometry using both cold (4°C) and warm (37°C) PD1 staining APC-PD1 (BD Biosciences, 558694) to visualize both intracellular/cycling and surface PD-1 and further confirmed by Sanger sequencing using the following primers (forward: CCCTTCCTCACCTCTCTCCA; reverse: CTGGAGCTCCTGATCCTGTG). The CRISPR gRNA used was CACGAAGCTCTCCGATGTGTTGG. The results from the genotyping are shown in Table S1.

Primary peripheral blood lymphocytes (PBLs) were isolated from buffy coats from healthy human volunteer donors, obtained from the State Hospital (Copenhagen, Denmark). Cells were cultivated in RPMI 1640 (Sigma, R5886) supplemented with 10% FBS (Sigma, F9665), 2 mM L-glutamine (Sigma, G7513), 2 mM penicillin and streptomycin (Sigma, P4333), as previously described (68). Primary peripheral blood mononuclear cells (PBMCs) were isolated by Histopaque-1077 density gradient centrifugation (Sigma, 10771) according to the manufacturer’s recommendations. To obtain PBLs, PBMCs were incubated for 1 h with washed IgG beads (Invitrogen, 11041), and monocytes were removed by magnet, as previously described (69). CD3^+^ and CD8^+^ T cells were purified from freshly purified PBMCs using the Dynabeads untouched human T cells kit (Invitrogen, 11344D) or the Dynabeads untouched human CD8^+^ T cells kit (Invitrogen, 11348D) according to the manufacturer’s recommendations. The purity of the isolated fractions was evaluated by flow cytometry to be above 90% for all donors.

### Bacterial strains and culture conditions

The clinical *S. aureus* isolates used in this study were SADR-1, SADR-2, SADR-3, SADR-4, and SADR-5, previously referred to as A9781, A9788, A9784, A9792, and A9798, respectively (27). The USA300 strain JE2 was purchased from the NARSA (Network of Antimicrobial Resistance in *Staphylococcus aureus*) program. USA300JE2*^spa_mut^* was obtained from the Nebraska Transposon Mutant Library (70), and USA300JE2*^clpP_mut^* was previously described. SADR-1*^rpoB_mut^* (SADR-1 with introduced mutation in *rpoB*-A477D), SADR-1*^clpP_mut^* (SADR-1 with introduced mutation in *clpP*-G94D), SADR-2*^clpP_rev_A^*, and SADR-2*^clpP_rev_B^* (two different clones of SADR-2 with reverted *clpP*-D94G) were previously described (4). All strains were cultivated in tryptic soy broth medium (TSB; Oxoid) under vigorous agitation (200 rpm) at 37°C. For solid medium, tryptic soy agar (TSA; Oxoid) was used. Strains and growth conditions has previously been described (26, 27).

### Preparation of live or UV-killed *S. aureus* and treatment of cells

*S. aureus* colonies from overnight cultures on TSA plates were inoculated in fresh TSB to OD600 ~0.03 and grown in Erlenmeyer flasks to early stationary phase (OD_600_ = 5–6, 5–7 h incubation). Bacteria were washed in PBS and resuspended in PBS to OD_600_ = 1. For stimulation with live *S. aureus*, bacterial solutions were added directly to human cell cultures. For preparing UV-killed *S. aureus* solutions, 5 mL of bacteria in PBS was transferred to petri dishes and subjected to pulsed UV radiation of 10,000 µJ/cm_2_ for 120 s (monochromatic wavelength of 254 nm; CL-1000 crosslinker; UVP, Cambridge, United Kingdom). Bacterial death was verified by plating on TSA plates and incubation at 37°C overnight (4). For the treatment of cells with live or UV-killed *S. aureus*, cells were seeded in supplemented RPMI-1640 medium at density of 6 × 10^5^ or 1 × 10^6^ cells/mL for Jurkat T cells and primary cells, respectively. Cells were treated with 50 µL UV-SA/mL cell suspension, PBS (untreated control), or 25 µL/mL prewashed CD3/CD28 dynabeads (Gibco, 11132D) and incubated at 37°C, 5% CO_2_ for indicated times.

### Flow cytometry

Cell-surface staining was done as previously described (68), except for data in S4a in which PD-1 staining was also performed directly in media and incubated at 37°C for 30 min to visualize both intracellular/cycling and surface PD-1. Cells were then washed twice in PBS, and for cells exposed to live *S. aureus,* these were further fixed using BD Cytofix Kit (BD Biosciences, 554714) prior to analysis. The antibodies used for the detection of surface expression were APC-PD1 (BD Biosciences, 558694), FITC-CD69 (BD Biosciences, 347823), PE-CD25 (BD Biosciences, 555431), APC-CD274 (eBioscience, 17–5983-41), PE-CD3 (BD Biosciences, 555333), PE-CD4 (BD Biosciences, 555347), and PE-CD8 (BD Biosciences, 555635). Appropriate isotype controls were purchased from BD Biosciences. Cell viability was assessed by staining with annexin V (BD Biosciences, 550474) and propidium iodide (Sigma, P4864). Staining was analyzed by the use of an Accuri C6 flow cytometer and CFlow software followed by analysis in the flow cytometry software program, FlowLogic version 8.2 (Inivai Technologies Pty, Australia). Gating was carried out on viable cells in forward-side scatter plots, and the grid was set according to the isotype controls (5%). Mean fluorescence intensity (MFI) values were presented as isotype MFI subtracted from MFI of specific staining. The MACSquant16 (Miltenyi Biotec Norden, Sweden) and the related MACSquantify software version 2.13 were used for the analysis of multicolor flow cytometry. The antibodies used for the detection of cell surface markers were PE-CD3 (Miltenyi Biotec, 130-113-139), Vio667-CD4 (Miltenyi Biotec, 130-115-200), APC-Vio770-CD8 (Miltenyi Biotec, 130-110-681), VioBlue-CD14 (Miltenyi Biotec, 130-110-524), PE-Vio770-CD19 (Miltenyi Biotec, 130-113-647), BV650-CD56 (BD Bioscience, 564057), FITC-CD16 (Thermo Fisher Scientific, MHCD1601), VioGreen-CD45 (Miltenyi Biotec, 130-110-638), PE-CD25 (Miltenyi Biotec, 130-113-286), PE-Vio770-CD69 (Miltenyi Biotech, 130-112-615), and VioBright 515-PD1 (Miltenyi Biotec,130-120-386). The LIVE/DEAD Fixable Yellow Dead Cell Stain Kit (Invitrogen, L34968) was used for the exclusion of dead cells, and all samples were fixed using the BD Cytofix Kit (BD Biosciences, 554714) before analysis. Appropriate isotype controls were purchased from the same vendor as the primary antibody. The gating strategies and panel setups are shown in Fig. S3a and b. Compensation was performed using the MACS compensation bead kits for human and mouse antibodies (Miltenyi Biotec, 130-104-693 and 130-097-900) following the manufacturer’s instructions and adjusted using appropriate fluorescence minus one (FMO) controls. For the evaluation of T-cell activation using surface marker expression, the grid was set according to the respective FMO controls to 5%. Proliferation of PBLs and purified T cells was analyzed by labeling with carboxyfluorescein succinimidyl ester (CFSE ; Molecular Probes, C34554) or CellTrace Violet (Molecular Probes, C34557), respectively, as previously described (71). Cells were reconstituted in PBS with 5% FBS (PBS + 5% FBS) at a density of 4 × 10^6^ cells/mL. CFSE or CellTrace Violet was diluted in PBS + 5% FBS to 10 µM and added to the cell suspension 1:1. Staining was carried out at RT for 5 min, under rotation, and protected from light. Cells were washed twice in RT PBS + 5% FBS and resuspended in standard supplemented RPMI media, counted, plated, and stimulated as described above.

### Calcium flux analysis

Calcium mobilization was measured using flow cytometry. Jurkat-PD1 or Jurkat-WT were diluted in Hanks balanced salt solution with Ca^2+^ and Mg^2+^ (HBSS) supplemented with 1% FBS to 6 × 10^6^ cells/mL and loaded with the Ca^2+^ indicator dye FuraRed (Molecular Probes, F3021; fluorescence decreases upon Ca^2+^ binding) at 37°C for 20 min. After two washing steps, the cells were diluted in HBSS and stored on ice until use. Before each run, the cells were equilibrated at 37°C for 5 min. FuraRed intensity was recorded continuously for 340 s using an Accuri C6 flow cytometer (BD Accuri). After 30 s baseline recording, stimuli (SADR-1, SADR-2, or controls) were added as indicated. An activating anti-CD3 antibody (clone: OKT3, eBioscience, 1 ug/mL) was used as a positive control, while the addition of pure HBSS was included as the negative control. Analysis was performed using FlowJo software (v. 10), and results are presented as the inverse FuraRed fluorescence intensity (normalized against the value at time_0), reflecting the relative cytosolic Ca^2+^ concentration, over time using GraphPad Prism software. For the comparison of separate experiments each data set was analyzed using the area under curve (AUC) function in GraphPad Prism for peaks above baseline (Y = 0) and ignoring peaks that are less than 10% above the baseline compared to the maximum peak height. The peak height of SADR or control treated samples was normalized to that of anti-CD3-stimulated samples for each experiment.

### Fluorescent labeling of UV-killed *S. aureus* and interaction with T cells

For assessing the interaction of *S. aureus* with PD-1 on the surface of Jurkat T cells, UV-killed *S. aureus* were fluorescently labeled using AlexaFluor647-conjugated succinimidyl ester (Molecular Probes, A-20006). UV-SA in PBS was pelleted by centrifugation at 10,000 *g*, 10 min at 4°C. Bacteria were resuspended in (original volume) sodium bicarbonate buffer (pH 8.5). SE-AF647 was added in a final concentration of 9 ng/µL, and the bacteria were incubated at agitation for 1 h at 4°C. UV-SA were washed in PBS and resuspended in the original volume of PBS. Jurkat-WT, Jurkat-PD1, or Jurkat-PD1-KO cells were seeded in supplemented RPMI-1640 medium at a density of 3 × 10^5^ cells/mL. Cells were treated with 50 µL/mL AF647-labeled *S. aureus* or PBS as control and incubated for 2–20 h at 37°C, 5% CO_2_. Unbound bacteria were washed away before analysis by flow cytometry (Accuri C6). Gating was done on viable cells, and the grid was placed according to the PBS-treated cells. PD-1-specific interaction is presented as percent interaction on Jurkat-PD1 or Jurkat-PD1-KO cells subtracted the percent interaction with Jurkat-WT cells.

### Interaction of UV-killed *S. aureus* with soluble PD1-fc receptor

UV-killed bacteria were investigated for direct binding to recombinant human PD1-Fc Chimera (R&D Systems, 1,086-PD-050). Recombinant IgG1-Fc Chimera (R&D, 110-HG-100) and recombinant human CD44-Fc Chimera (R&D, 3,660 CD-050) were used as controls. The Fc-chimeric proteins were labeled with Zenon Alexa Fluor 647 human IgG labeling kit (Molecular Probes, Z-25408) prior to the binding assay; 50 µL UV-SA suspension in PBS was pelleted by centrifugation at 10,000 *g*, 10 min at 4°C. Bacteria were resuspended in 42 µL PBS with rabbit IgG (Sigma, I8140) (diluted 1:500); and 8 µL AF647-labeled Fc-chimeric protein suspension was added to each bacterial sample and incubated at agitation for 30 min at 4°C. Samples were washed twice in PBS including rabbit IgG (1:500). Samples were resuspended in PBS, and the interaction was assessed by flow cytometry (Accuri C6). Gating was done according to a background of 1% in a PBS sample at a threshold of 5,000 in the forward scatter. The grid was placed for every unstained *S. aureus* strain in PBS.

### Microscopy

For images on confocal microscopy, SADR-2 was labeled with SE-AF647 as described above. Jurkat T cells were plated in supplemented RPMI-1640 medium at a density of 3 × 10^5^ cells/mL; cells were treated with 50 µL/mL AF-SADR2 or PBS as control and incubated at 37°C, 5% CO_2_, for 16–20 h before analysis. Jurkat-WT cells were washed twice in PBS supplemented with 2% FBS prior to staining with AF488 conjugated CD45 (Biolegend, 304019) at 4°C for 30 min. Jurkat-PD1 cells were washed twice in PBS supplemented with 2% FBS. All samples were resuspended in PBS for analysis. Confocal images were taken using a Carl Zeiss LSM780 confocal system with a Plan-Apochromat 63×/1.4 oil-immersion objective. The 505 nm and 633 nm lasers were used to excite GFP (in Jurkat-PD1 cells), AF488-CD45 and SE-AF647, respectively. Fluorescent signals were collected using a fMBS 405/505 c beam splitter and acquired in the same track to minimize cell movement during acquisition. Images were processed using the software Zen Blue version 2.3. The cell surface (green color) was visualized with AF488-CD45 antibody in Jurkat-WT cells and GFP (coupled to PD-1) expression in Jurkat-PD1 cells. AF647-labeled *S. aureus* appeared in magenta color.

### Design, production, and quality control of anti-PD1-IgG1 (IgG1-αPD1) blocking antibody

The variable region for the anti-PD1 IgG1 variant is based on the sequence of Nivolumab (Bristoll Meyers Squibb) found in the IMGT database (72). The antibody sequences of the variable heavy (V_H_) and light (V_L_) regions were cloned into pcDNA3.1 with Neomycin and Zeocin resistance (Invitrogen) containing sequences for the human constant domains of the IgG1 κ light chain (LC) (UniProt P01834) and heavy chain (HC) (UniProt P01857), respectively (73). Both plasmids were transiently co-transfected in CHO-S cell lines at a HC:LC ratio of 3:2 followed by the harvest of cell culture supernatants containing the antibody at 96–148 h after transfection. Antibodies were purified using MabSelect Xtra or MabSelect SuRe columns (GE-Healthcare), depending on the scale of the production. Endotoxin levels were below 0.3 EU/mL in antibody preparations of concentration used in the T-cell cultures as determined by the Limulus Amoebocyte Lysate (LAL) endotoxin detection assay (Pierce). The affinity of the engineered IgG1-αPD1 to recombinant human (rh)PD-1 (Acro Biosystems, PD1-H5221) was verified and compared to Nivolumab (IgG4-αPD1; Opdivo, Evidentic) using Biolayer interferometry (BLI) using AHC biosensors (ForteBio) on an OctetRed96 system (ForteBio) (74). The following steps were used in the kinetic assay: (i) baseline = PBST (PBS pH 7.4 + 0.02% Tween 20 +0.1% BSA), 60 s, 1,000 rpm; (ii) loading = 2.5 µg/mL IgG1- or IgG4-αPD1, 120 s, 1,000 rpm; (iii) baseline 2 = PBST, 60 s, 1,000 rpm; (iv) association = 100–1.6 nM rhPD-1, 300 s, 1,000 rpm; (v) dissociation = PBST, 300 s, 1,000 rpm. K_D_ values were determined by a steady-state analysis of data after alignment to baseline 2 and subtraction of the reference sensor exposed to PBST only. Binding of IgG1- and IgG4-αPD1 to Jurkat-WT, Jurkat-PD1, and Jurkat PD1 KO cells was determined using flow cytometry. Initially, IgG1- and IgG4-αPD1 were fluorescently labeled using the Zenon human IgG labeling kit AF647 (Invitrogen, Z25408) following the manufacturer’s protocol. After blockade of excess labeling reagent, the antibodies were diluted serially from 6 to 0.003 ug/mL to make a titration curve and incubated with the cells at 4° for 10 min. Cells were then washed twice in PBS and analyzed on a MACSquant16 flow cytometer (Miltenyi Biotec).

### Effect of *in vitro* PD-1 blockade on T-cell activation

For the evaluation of the effect of PD1 blockade on *S. aureus*-induced T-cell activation *in vitro,* purified human CD3^+^ or CD8^+^ T cells were seeded in supplemented RPMI-1640 medium at a density of 1 × 10^6^ cells/mL. After seeding, the cells were treated with 1 µg/mL IgG1- or IgG4-αPD1 or Ultra-LEAF purified human IgG1 (Biolegend, 403502) or Ultra-LEAF purified human IgG4 (Biolegend, 403702) as isotype controls in combination with 50 µL UV-SA/mL cell suspension, PBS (untreated control), or 5 µL/mL T cell TransAct (CD3/CD28) as a positive control (Miltenyi Biotec, 130–111-160). After treatment, the cells were incubated at 37°C, 5% CO_2_ for 5 days before harvest of the cell culture supernatants for cytokine analysis and preparation of cells for flow cytometry.

### Quantification of cytokines in culture supernatants

Levels of IL-2 and IFN-γ from human primary lymphocytes were assessed by enzyme-linked immunosorbent assay (ELISA) on day 1 and day 6 after treatment with *S. aureus*. Standard ELISA was used for measuring human IL-2 (R&D Systems, DY202) and human IFN-γ (R&D Systems, DY285B) in harvested culture supernatants. Samples were run on a BioTek PowerWave instrument and analyzed using KC4v3.0 software.

Levels of GM-CSF, IFN-γ, IL-2, IL-4, IL-9, IL-10, IL-13, IL-17A, IL-17E/IL-25, IL-17F, IL-21, IL-22, MIP-3α, and TNF-α were measured in supernatants from CD3^+^ and CD8^+^ T cells by V-PLEX (K151QQD) or U-PLEX (K15093K) using chemiluminescence-based assays from Meso Scale Discovery (MSD, Gaithersburg, MD, USA). Analyses were done using a QuickPlex SQ 120 instrument (MSD) and DISCOVERY WORKBENCH 4.0 software.

### Statistical analysis

Data preparation and statistical analysis (see figure legends for details) were performed using the software GraphPad Prism, version 9.1.0 (Graphpad Software, USA). Data are presented as mean ± s.e.m. (standard error of mean), and level of statistical significance was determined by *P*-value < 0.05.

## SUPPLEMENTAL MATERIAL

Fig. S1 mbio.01349-23-s0001.tif Clinical *S. aureus* isolates and T cell activation.

Fig. S2 mbio.01349-23-s0002.tif Mutation in *rpoB* is not responsible for the observed T cell suppressing phenotype of *S. aureus*.

Fig S3 mbio.01349-23-s0003.tif
*S. aureus* directly activates human T cells.

Fig. S4 mbio.01349-23-s0004.tif PD-1 expression and fluorescent labelling of *S. aureus*.

Fig. S5 mbio.01349-23-s0005.tif PD-1 blockade rescues CD8^+^ T cell functions after SADR-2 stimulation in CD3^+^ cultures only.

Fig. S6 mbio.01349-23-s0006.tif PD-1 blockade does not rescue T cell functions after SADR-2 stimulation in pure CD8^+^ T cell cultures.

Fig. S7 mbio.01349-23-s0007.tif Post-transcriptional induction of PD-1 on Jurkat T cells by *S. aureus* and effect of *S. aureus*-carrier status on T cell activation.

Supplemental text mbio.01349-23-s0008.docx Supplemental figure legends and methods.

Table S1 mbio.01349-23-s0009.docx PD1 KO cells.

ASM does not own the copyrights to Supplemental Material that may be linked to, or accessed through, an article. The authors have granted ASM a non-exclusive, world-wide license to publish the Supplemental Material files. Please contact the corresponding author directly for reuse.
