# Supplementary figures and images for: Clinical Staphylococcus aureus inhibits human T-cell activity through interaction with the PD-1 receptor
Source: mBio. 2023 Oct 5;14(5):e01349-23. doi: 10.1128/mbio.01349-23 (PMC10653905; doi:10.1128/mbio.01349-23)

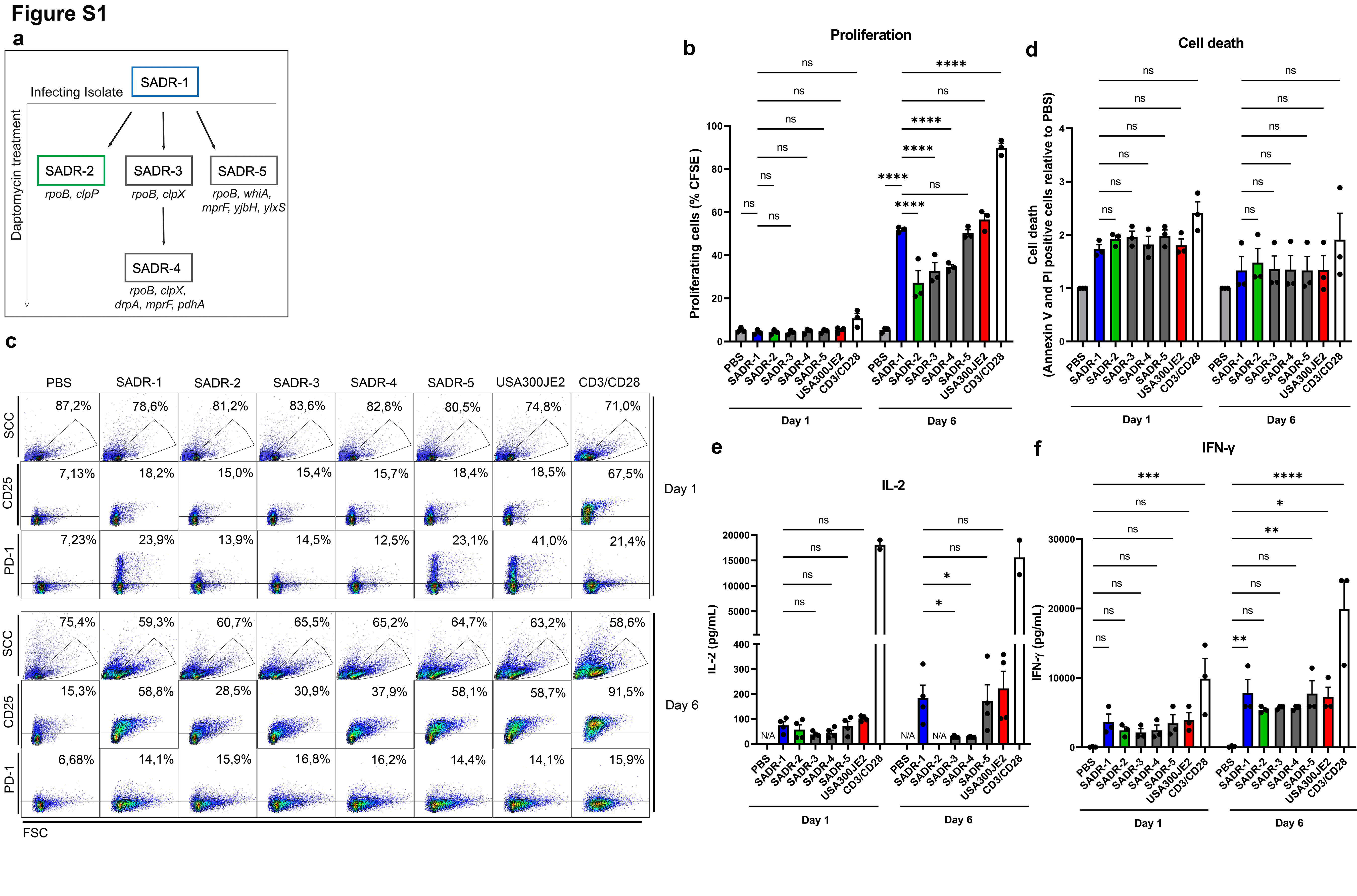

Supplement: Fig. S1 — Clinical S. aureus isolates and T cell activation. [file mbio.01349-23-s0001.tif]

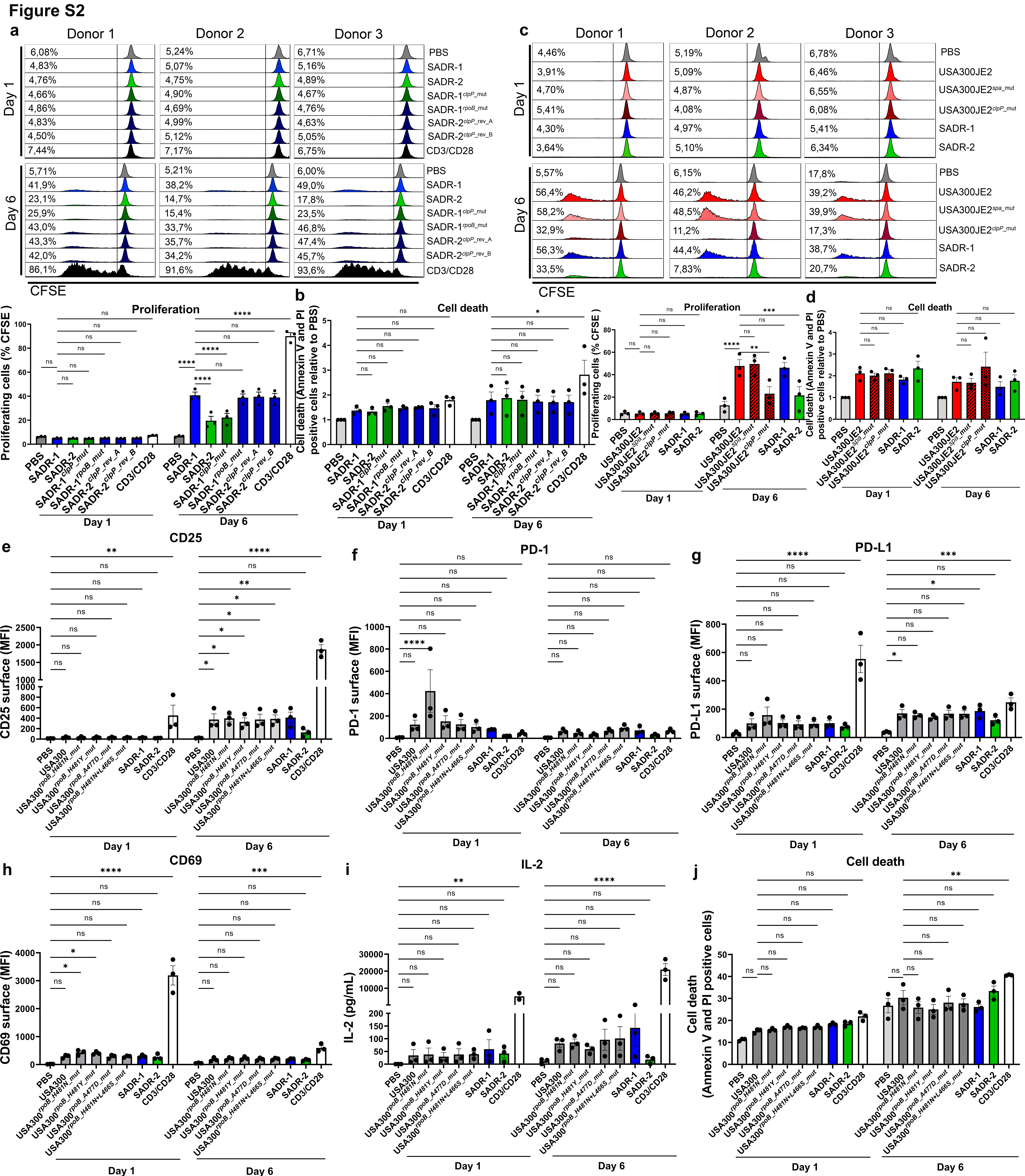

Supplement: Fig. S2 — Mutation in rpoB is not responsible for the observed T cell suppressing phenotype of S. aureus. [file mbio.01349-23-s0002.tif]

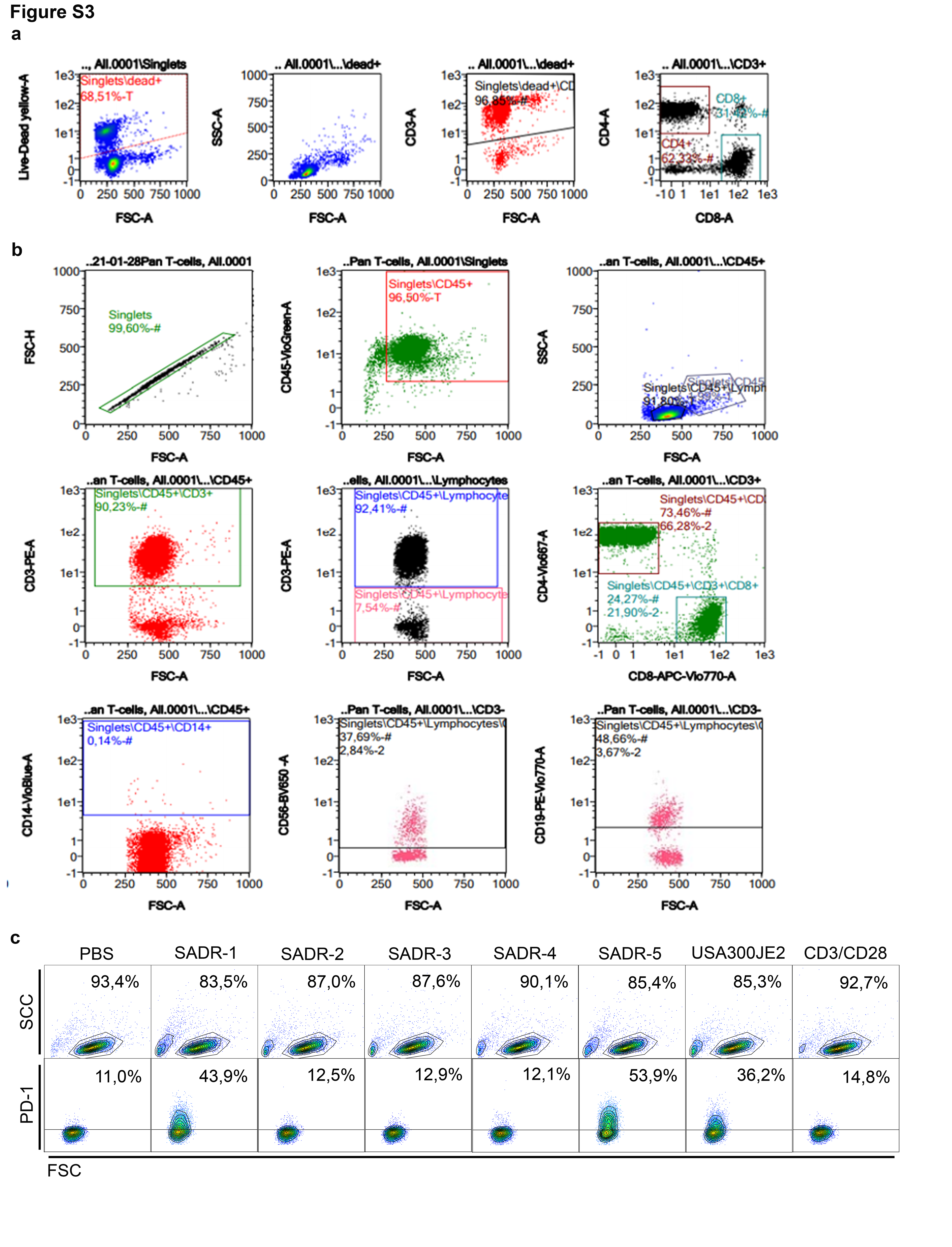

Supplement: Fig S3 — S. aureus directly activates human T cells. [file mbio.01349-23-s0003.tif]

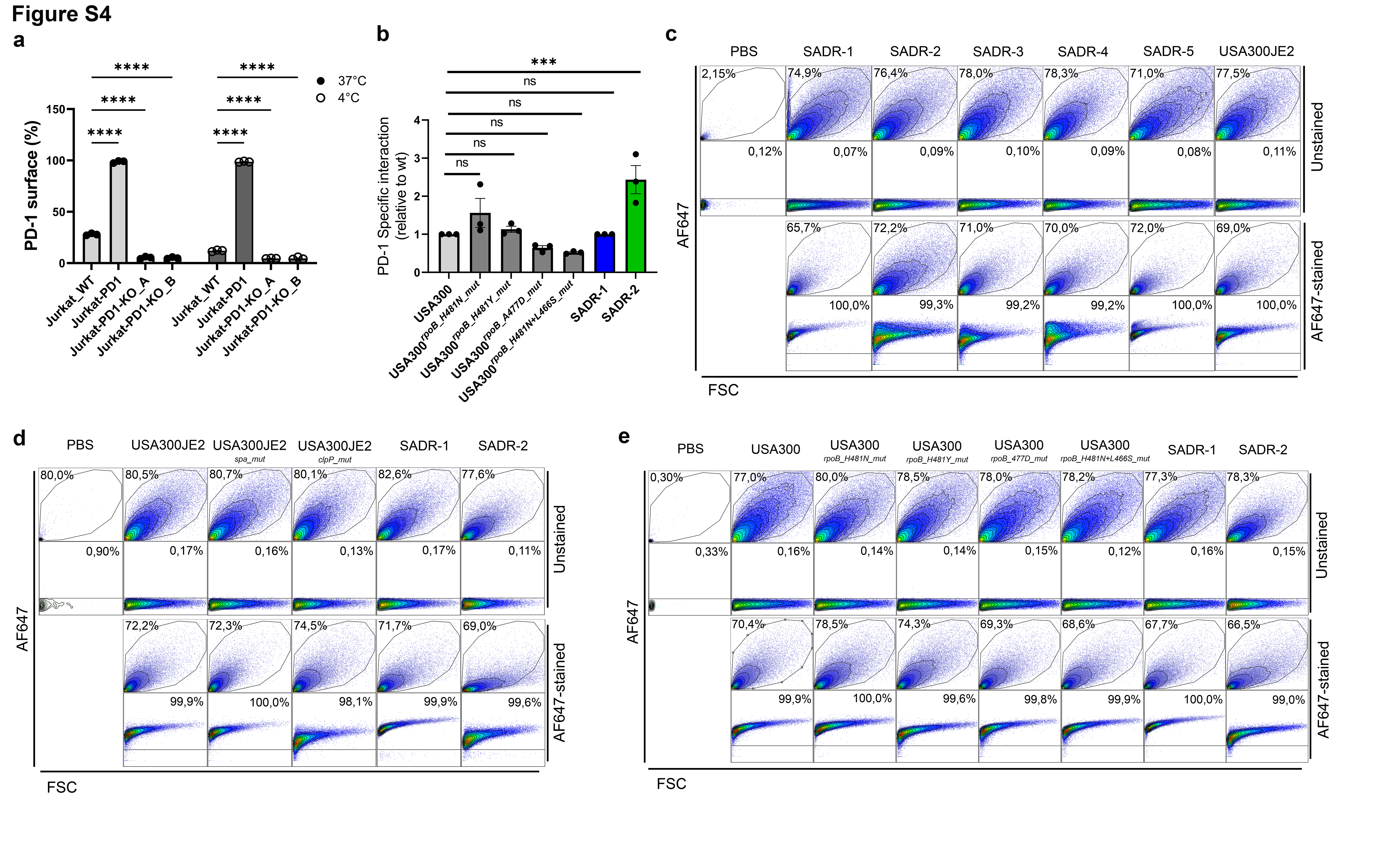

Supplement: Fig. S4 — PD-1 expression and fluorescent labelling of S. aureus. [file mbio.01349-23-s0004.tif]

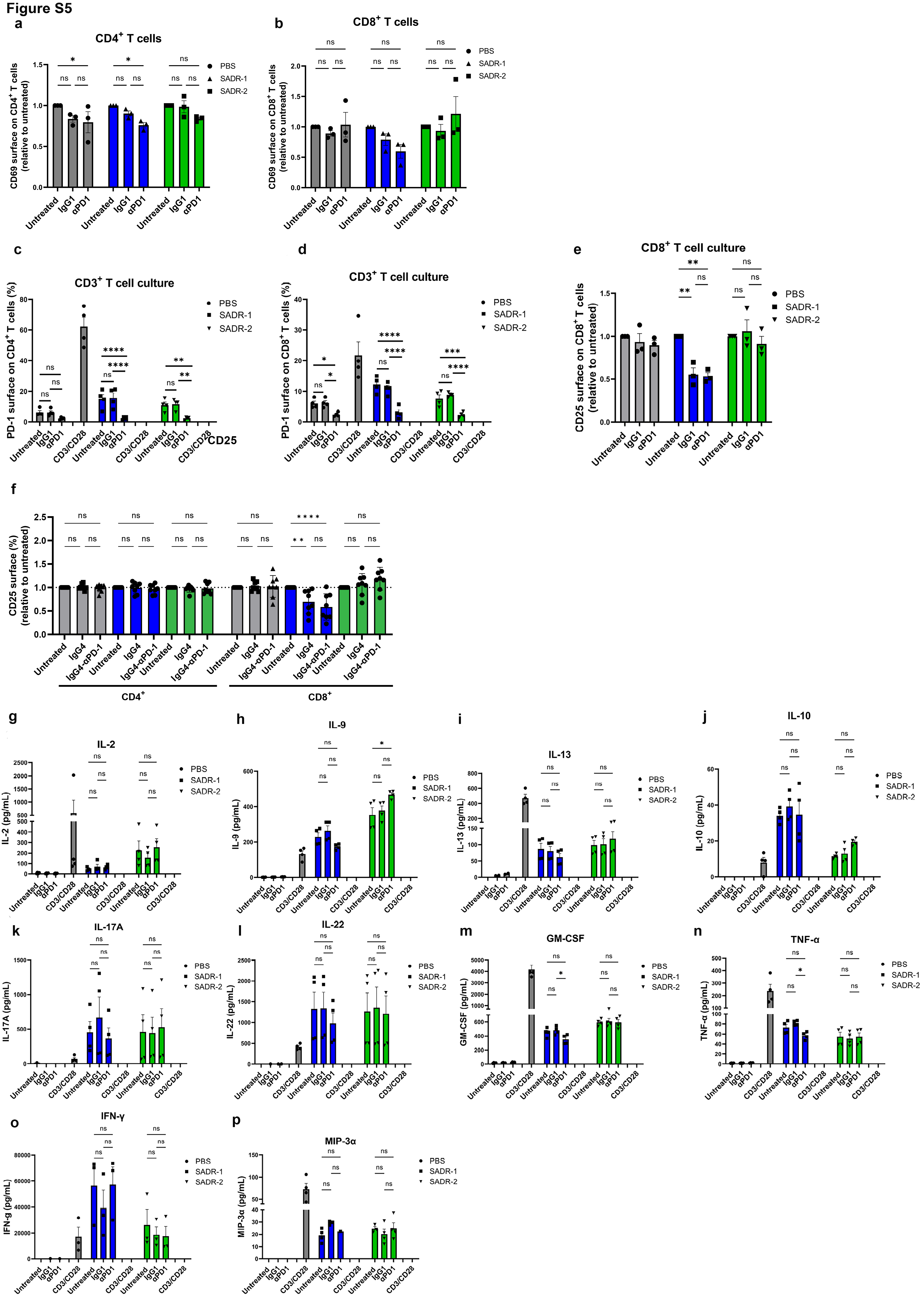

Supplement: Fig. S5 — PD-1 blockade rescues CD8+ T cell functions after SADR-2 stimulation in CD3+ cultures only. [file mbio.01349-23-s0005.tif]

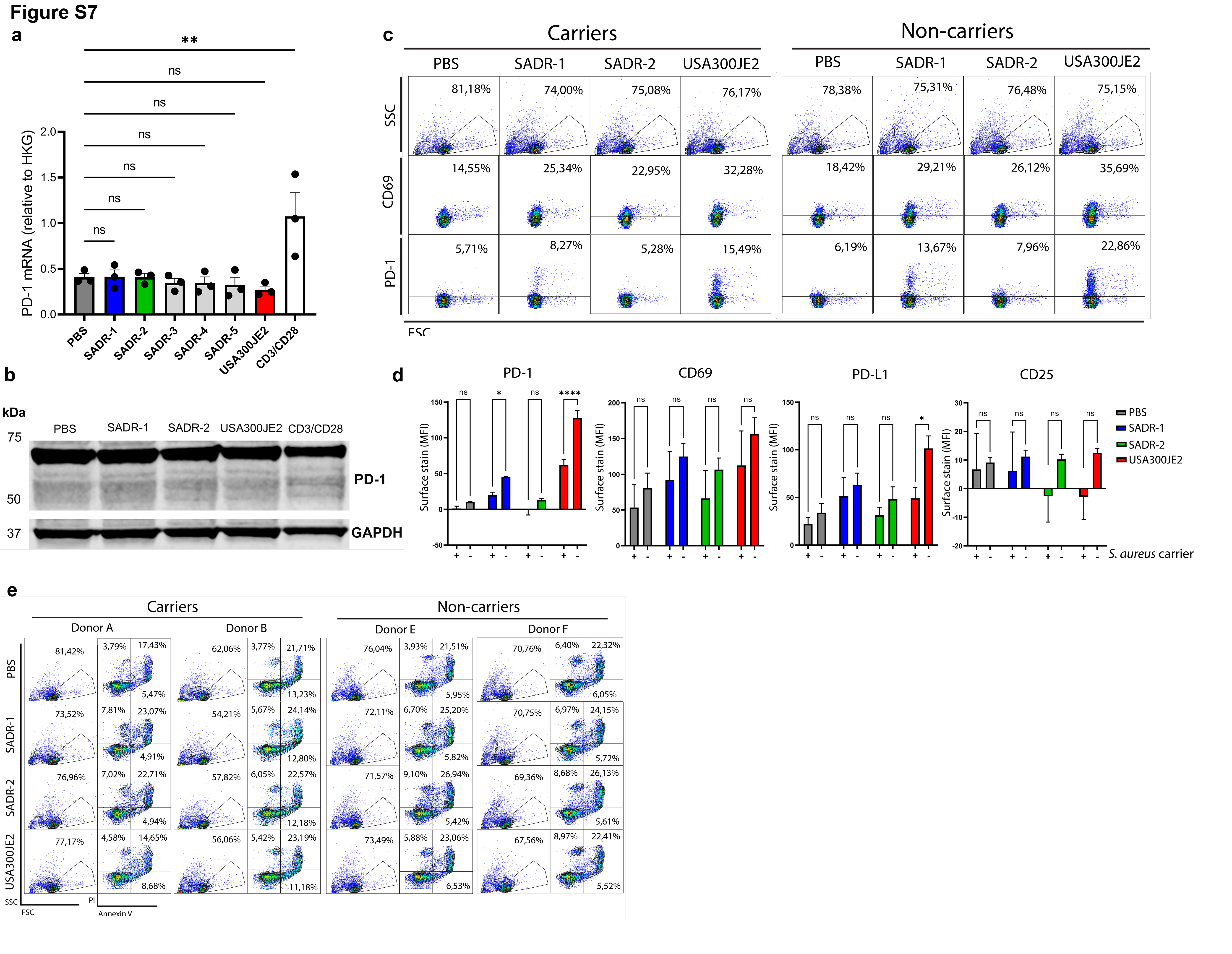

Supplement: Fig. S7 — Post-transcriptional induction of PD-1 on Jurkat T cells by S. aureus and effect of S. aureus-carrier status on T cell activation. [file mbio.01349-23-s0007.tif]
