# Supplementary material for: Clinical Staphylococcus aureus inhibits human T-cell activity through interaction with the PD-1 receptor
Source: mBio. 2023 Oct 5;14(5):e01349-23. doi: 10.1128/mbio.01349-23 (PMC10653905; doi:10.1128/mbio.01349-23)
Supplement: Supplemental text — Supplemental figure legends and methods. [file mbio.01349-23-s0008.docx]

**Clinical *Staphylococcus aureus* inhibits human T cell activity through interaction with the PD-1 receptor**

*Maiken Mellergaard^1^*^*^*, Sarah Line Skovbakke^,^*^2*^*, Stine Dam Jepsen^1^, Nafsika Panagiotopoulou^1^, Amalie Bøge Rud Hansen^1^, Weihua Tian*^2^*, Astrid Lund^1^, Rikke Illum Høgh^1^, Sofie Hedlund Møller^1^, Romain Guérillot^3^, Ashleigh S. Hayes^3^, Lise Tornvig Erikstrup*^4^*, Lars Andresen^1^, Anton Y. Peleg*^5,6,7^*, Anders Rhod Larsen*^8^*, Timothy P. Stinear*^3^*, Aase Handberg^9,10^, Christian Erikstrup^11^, Benjamin P. Howden*^3^*, Steffen Goletz^2,#^, Dorte Free^12^ and Søren Skov^1, #,^**

**^1^**Laboratory of immunology, Experimental Animal Models, Department of Veterinary and Animal Sciences, Faculty of Health and Medical Sciences, University of Copenhagen, Denmark.

^2^Biotherapeutic Glycoengineering and Immunology, DTU Bioengineering, Department of Biotechnology and Biomedicine, Technical University of Denmark, Lyngby, Denmark.

^3^Department of Microbiology and Immunology, University of Melbourne, at the Peter Doherty Institute for Infection and Immunity, Victoria, 3000, Australia.

^4^Department of Clinical Microbiology, Aarhus University Hospital, Aarhus, Denmark.

^5^Department of Microbiology, Monash University, Melbourne, Victoria, Australia.

^6^Infection Program, Monash Biomedicine Discovery Institute, Department of Microbiology, Monash University, Melbourne, Victoria, Australia.

^7^Centre to Impact Antimicrobial Resistance, Monash University, Melbourne, Victoria, Australia.

^8^Statens Serum Institute, Microbiology and Infection Control, Copenhagen, Denmark.

**^9^**Department of Clinical Biochemistry, Aalborg University Hospital, North Denmark Region, Denmark.

^10^Department of Clinical Medicine, Aalborg University, Aalborg, Denmark.

^11^Department of Clinical Immunology, Aarhus University Hospital, Aarhus, Denmark.

^12^Food Safety and Zoonosis, Department of Veterinary and Animal Sciences, Faculty of Health and Medical Sciences, University of Copenhagen, Denmark.

* Authors contributed equally to this work/shared first authorship. Author order was determined by agreement considering that MM initiated the project. # Shared senior authorship

*Corresponding author: Søren Skov, University of Copenhagen, Laboratory of Immunology, Ridebanevej 9, DK-1870 Frederiksberg, Denmark. Phone: +4535333126; E-mail: sosk@sund.ku.dk

## Running Title: ClpP mutant S. aureus inhibits T cells

**Supplementary methods**

***Quantitative real-time PCR***

Whole-cell mRNA was isolated from Jurkat T cells treated with UV-SA for 8 h using TRizol reagent (Invitrogen, 15596026) and Direct-zol RNA Miniprep (Zymo research, R2050S). The mRNA was converted to cDNA by iScript Advanced cDNA Synthesis kit, (BioRad, 172-5038). RPLP0 (ribosomal protein, large P0) was used as housekeeping gene. Primer-sequences for PCR were: RPLP0: Forward_ RPLP0_119: 5´-CCTCGTGGAAGTGACATCGT-´3 and Reverse_ RPLP0_433: 5´-CATTCCCCCGGATATGAGGC-´3, PD1; Forward_PD1_1538: 5´- GGAAACCCCTCCACCTTTA -´3 and Reverse_PD1_1625: 5´- TCTGCCTGCCCGCTTACT -´3, previously published[^1^](#_ENREF_1). Primers were purchased from Eurofins MWG Operon. PCR amplification was performed using QuantiNova SYBR Green master mix with low ROX (1076685; Quiagen) on a Bio-Rad CFX apparatus and analyzed using Bio-Rad CFX Maestro. The relative expression levels of PD1 were normalized to expression of the housekeeping gene.

***Western blot***

Whole cell lysates were prepared in 1 % Triton X-100, 25mM Tris-HCl (pH 7,4), 150mM NaCl, 5mM EDTA, 1 % SDS including Halt^TM^ Protease and Phosphatase Inhibitor Cocktail (Thermo Scientific, 78440). Lysates were sonicated and then resolved by SDS-PAGE electrophoresis, as previously described[^2^](#_ENREF_2). Antibodies for western blotting were anti-PD-1 (Invitrogen, PA5-20350) and anti-GAPDH (Cell Signaling Technology, 2118), IRDye 800CW Donkey anti-Rabbit IgG Secondary antibody (Licor Biosciences, 926-32213), and IRDye 680LT Donkey anti-Mouse IgG Secondary antibody (Licor Biosciences, 926-68022). Protein staining was visualized by Odyssey Fc Imager (LI-COR Biosciences, Cambridge, UK).

***PBLs from donors of known S. aureus-carrier status***

Primary PBLs were isolated from donors of known *S. aureus* carrier status. PBLs were isolated and cultivated as described in Materials and Methods and ethical approval, informed consent from donors, and determination of *S. aureus* carrier status were described in the Danish blood donor *Staphylococcus aureus* carriage study[^3^](#_ENREF_3).

***Bacterial strains***

USA300 strain NRS384 (USA300), USA300*^rpoB_H481N_mut^* (USA300 strain NRS384 with introduced mutation in *rpoB*-H481N), USA300*^rpoB_H481Y_mut^* (USA300 strain NRS384 with introduced mutation in *rpoB*-H481Y), USA300*^rpoB_A477D_mut^* (USA300 strain NRS384 with introduced mutation in *rpoB*-A477D), and USA300*^rpoB_H481N+L466S_mut^* (USA300 strain NRS384 with introduced mutation in *rpoB*-H481N+L466S) were previously described[^4^](#_ENREF_4). Bacteria were cultivated as described in Materials and Methods.

**References**

1. Pan, T. *et al.* Notch Signaling Pathway Was Involved in Regulating Programmed Cell Death 1 Expression during Sepsis-Induced Immunosuppression. *Mediators of inflammation* **2015**, 539841 (2015).

2. Jensen, H., Hagemann-Jensen, M., Lauridsen, F. & Skov, S. Regulation of NKG2D-ligand cell surface expression by intracellular calcium after HDAC-inhibitor treatment. *Molecular Immunology* **53**, 255-264 (2013).

3. Erikstrup, L.T. *et al.* Cohort description: The Danish Blood Donor Staphylococcus aureus Carriage Study. *Clin Epidemiol* **11**, 885-900 (2019).

4. Guérillot, R. *et al.* Convergent Evolution Driven by Rifampin Exacerbates the Global Burden of Drug-Resistant &lt;span class=&quot;named-content genus-species&quot; id=&quot;named-content-1&quot;&gt;Staphylococcus aureus&lt;/span&gt. *mSphere* **3**, e00550-00517 (2018).

5. Mellergaard, M. *et al.* Staphylococcus aureus induces cell-surface expression of immune stimulatory NKG2D ligands on human monocytes. *J Biol Chem* (2020).

**Supplementary figures**

**Figure S1: Clinical *S. aureus* isolates and T cell activation.**

**a**, Clinical isogenic *S. aureus* strains SADR-1, SADR-2, SADR-3, SADR-4, and SADR-5. Arrows represent acquired SNPs and decreasing daptomycin susceptibility that the isolates have gained during development. SADR-1 (infecting MRSA isolate), SADR-2 and SADR-3 (decreased daptomycin susceptibility), SADR-4 and SADR-5 (daptomycin non-susceptible) (**a**), previously described[^5^](#_ENREF_5). **b**-**f**, Human PBLs were treated with PBS, SADR1-5, USA300JE2, or CD3/CD28-beads and analyzed on day 1 and 6 for proliferation (**b**) surface CD25 and PD-1 (**c**), Annexin V and PI (**d**), secretion of IL-2 (**e**), and IFN-γ (**f**). Dotplots are representative of three independent experiments depicting %-positive cells and results in bargraphs are pooled from three donors (n=3) showing mean ± s.e.m for %-positive cells in **b**, Annexin V and PI positive cells relative to control cells (PBS) in **d**, and cytokine concentration (pg/mL) in **e**-**f**. Statistical analysis was performed by two-way ANOVA with Dunnett´s multiple comparisons test in **b**-**f**. *p<0.05, **p<0.01, ***p<0.001, ****p<0.0001.

**Figure S2: Mutation in *rpoB* is not responsible for the observed T cell suppressing phenotype of *S. aureus*. a**-**j**, Human PBLs were analyzed on day 1 and 6 after stimulation with **a-b**: PBS, SADR-1, SADR-2, SADR-1*^rpoB_mut^*, SADR-1*^clpP_mut^*, SADR-2*^clpP_rev_A^*, SADR-2*^clpP_rev_B^*, or CD3/CD28-beads and analyzed for proliferation (CFSE) (**a**), and cell death (Annexin V and PI) (**b**), **c**-**d**: PBS, USA300JE2, USA300JE2*^spa_mut^*, USA300JE2*^clpP_mut^*, SADR-1, or SADR-2 and analyzed for proliferation (CFSE) (**c**), and cell death (Annexin V and PI) (**d**), or **e**-**j**: USA300, USA300*^rpoB_H481N_mut^*, USA300*^rpoB_H481Y_mut^,* USA300*^rpoB_A477D_mut^,* USA300*^rpoB_H481N+L466S_mut^*, SADR-1 or SADR-2 and analyzed for surface expression of CD25 (**e**), PD-1 (**f**), PD-L1 (**g**), CD69 (**h**), secretion of IL-2 (**i**), and cell death (Annexin V and PI) (**j**). Histograms are representative of three independent experiments depicting %-CFSE positive cells and results in bargraphs are pooled from three donors (n=3) showing mean ± s.e.m for %-CFSE positive cells in **a** and **c**, Annexin V and PI positive cells in **b**, **d**, and **j**, pg/mL in **i**, and mean fluorescence intensity (MFI) in **e**-**h**. Statistical analysis was performed by two-way ANOVA with Dunnett´s multiple comparisons test for **b** and **d**-**j.** *p<0.05, **p<0.01, ***p<0.001, ****p<0.0001.

**Figure S3: *S. aureus* directly activates human T cells.**

**a**-**b**, Human CD3^+^ T cells were purified from buffy coats. Gating was done on viable, CD3^+^-positive cells, and subsequently CD4^+^ and CD8^+^ positive cells (**a**) and purity of culture was tested by co-staining with CD14, CD56, and CD19 (**b**). Human Jurkat T cells (JTag) were analyzed on day 1 after stimulation with PBS, SADR-1-5, USA300JE2, or CD3/CD28 and analyzed for expression of PD-1 surface expression (**c**). Dotplots are representative of three independent experiments (n=3).

**Figure S4: PD-1 expression and fluorescent labelling of *S. aureus*.**

**a**-**b**, Jurkat-WT, Jurkat-PD1, and Jurkat-PD1-KO (two different clones A and B) T cells were analyzed for expression of PD-1 by flow cytometry (**a**). Jurkat-PD1 and Jurkat-WT T cells were treated with PBS or AF647-labelled *S. aureus* strains. PD-1-specific interaction was analyzed after 14-20 h by flow cytometry for USA300, USA300*^rpoB_H481N_mut^*, USA300*^rpoB_H481Y_mut^,* USA300*^rpoB_A477D_mut^,* USA300*^rpoB_H481N+L466S_mut^*, SADR-1 or SADR-2 (**b**). **c**-**e**: Verification of AF647-labeling of *S. aureus* for SADR1-5, and USA300JE2 (c), USA300JE2, USA300JE2*^spa_mut^*, USA300JE2*^clpP_mut^*, SADR-1, and SADR-2 (**d**), and USA300, USA300*^rpoB_H481N_mut^*, USA300*^rpoB_H481Y_mut^,* USA300*^rpoB_A477D_mut^,* USA300*^rpoB_H481N+L466S_mut^*, SADR-1 or SADR-2 (e). Dotplots are representative of two independent staining procedures (n=2). Data represent mean ± s.e.m. (n=3) of % PD-1 surface expression in **a** and PD-1-specific interaction relative to WT (USA300 and SADR-1, respectively) in **b**. Statistical analysis was performed by two-way ANOVA with Dunnett´s multiple comparisons test for **a**, and one-way ANOVA with Dunnett´s multiple comparisons test in **b.** ***p<0.001, ****p<0.0001.

**Figure S5: PD-1 blockade rescues CD8^+^ T cell functions after SADR-2 stimulation in CD3^+^ cultures only.**

**a**-**p**, Purified human CD3^+^ T cells (**a-d** and **f-p**) or CD8^+^ T cell culture (**e**) were treated with PBS, SADR-1, or SADR-2 alone or combined with anti-PD-1 block (αPD1 or IgG4-αPD1) and analyzed on day 5 for surface expression of PD-1 gated on CD4^+^ T cells (**a**, **c**, and **f**) and on CD8^+^ T cells (**b**, **d**, and **f**). Purified human CD8^+^ T cells were treated with PBS, SADR-1, or SADR-2 alone or combined with anti-PD-1 block (αPD1) and analyzed on day 5 for surface expression of CD25 relative to untreated controls (**e**). Secretion of cytokines (pg/mL) from CD3^+^ T cells treated with PBS, SADR-1, or SADR-2 alone or combined with anti-PD-1 block (αPD1) and analyzed on day 5 for IL-2 (**g**), IL-9 (**h**), IL-13 (**i**), IL-10 (**j**), IL-17A (**k**), IL-22 (**l**), GM-CSF (**m**), TNF-α (**n**), IFN-γ (**o**), and MIP-3α (**p**). The data are shown as mean ± s.e.m (n=3-4), presenting surface expression relative to untreated controls in **a-b** and **f**, % surface expression in **c**-**e**, and pg/mL in **g**-**p**. Statistical analysis was performed by two-way ANOVA with Tukey´s multiple comparisons test. *p<0.05, **p<0.01, ***p<0.001, ****p<0.0001.

**Figure S6: PD-1 blockade does not rescue T cell functions after SADR-2 stimulation in pure CD8^+^ T cell cultures.**

**a**-**g**, Purified human CD8^+^ T cells were treated with PBS, SADR-1, or SADR-2 alone or combined with anti-PD-1 block (αPD1) and analyzed on day 5 for secretion of (pg/mL) IL-2 (**a**), IL-9 (**b**), TNF-α (**c**), IL-22 (**d**), IL-13 (**e**), IFN-γ (**f**), and GM-CSF (**g**). The data are shown as mean ± s.e.m (n=2-3). Statistical analysis was performed by two-way ANOVA with Tukey´s multiple comparisons test in **a**-**g**.

**Figure S7: Post-transcriptional induction of PD-1 on Jurkat T cells by *S. aureus* and effect of *S. aureus*-carrier status on T cell activation.**

**a**-**b**, Human Jurkat T cells (JTag) were analyzed 8 h after stimulation with PBS, SADR-1-5, USA300JE2, or CD3/CD28 and for expression of PD-1 mRNA expression by qPCR (**a**) and whole cell protein by western blotting (**b**). Data represent mean ± s.e.m. of PD-1 mRNA relative to housekeeping gene (HKG) and relative to PBS for three independent experiments (n=3). Westernblot is representative of three independent experiments (n=3). **c**-**e**, Purified human PBLs from donors of known *S. aureus*-carrier status were treated with PBS, SADR-1, SADR-2, or USA300JE2 and analyzed on day 1 for surface expression of PD-1, CD69, CD25, and PD-L1 (**c**-**d**) or Annexin V and PI (**e**). Dotplots are representative of three independent experiments and results in bargraphs are pooled from carriers (n=4) and non-carriers (n=3) representing mean fluorescence intensity (MFI) ± s.e.m. Statistical analysis was performed by one-way ANOVA with Dunnett´s multiple comparisons test in **a** and two-way ANOVA with Sidak´s multiple comparisons test in **d**. *p<0.05, **p<0.01, ****p<0.0001.
