# Supplementary material for: Clinical Staphylococcus aureus inhibits human T-cell activity through interaction with the PD-1 receptor
Source: mBio. 2023 Oct 5;14(5):e01349-23. doi: 10.1128/mbio.01349-23 (PMC10653905; doi:10.1128/mbio.01349-23)
Supplement: Table S1 — PD1 KO cells. [file mbio.01349-23-s0009.docx]

| **Clone** | **Targeted genes** | **InDel** | **Alignment** | **Contribution** |
| --- | --- | --- | --- | --- |
| E10 | PDCD1 WT |  | CACGAAGCTCTCCGATGTGTTGG |  |
|  |  |  |  |  |
|  | KO-alle1 | +11 bp | CACGAAGCTCTCCGATGNNNNNNNNNNNTGTTGG | 25% |
|  | KO-alle2 | +1 bp | CACGAAGCTCTCCGATGNTGTTGG | 24% |
|  | KO-alle3 | +3 bp | CACGAAGCTCTCCGATGNNNTGTTGG | 16% |
|  | KO-alle4 | 0 bp | CACGAAGCTCTCCGATGTGTTGG | 5% |
|  | KO-alle5 | +2 bp | CACGAAGCTCTCCGATGNNTGTTGG | 3% |
|  | KO-alle6 | +9 bp | CACGAAGCTCTCCGATGNNNNNNNNNTGTTGG | 2% |
|  | KO-alle7 | -2 bp | CACGAAGCTCTCCGATG--TTGG | 1% |
|  |  |  |  |  |
| H10 | PDCD1 WT |  | CACGAAGCTCTCCGATGTGTTGG |  |
|  |  |  |  |  |
|  | KO-alle1 | +11 bp | CACGAAGCTCTCCGATGNNNNNNNNNNNTGTTGG | 33% |
|  | KO-alle2 | +8 bp | CACGAAGCTCTCCGATGNNNNNNNNTGTTGG | 27% |
|  | KO-alle3 | +3 bp | CACGAAGCTCTCCGATGNNNTGTTGG | 15% |
|  | KO-alle4 | +10 bp | CACGAAGCTCTCCGATGNNNNNNNNNNTGTTGG | 3% |
|  | KO-alle5 | +7 bp | CACGAAGCTCTCCGATGNNNNNNNTGTTGG | 2% |
|  |  |  |  |  |

**Supplementary Table 1:** Sequence analysis of PDCD1 KO in Jurkat cells

Nucleic acids UNDERLINED are the gRNA targeting sequence, nucleic acids in RED are the PAM sequence, Nucleic acids in N are insertions, and nucleic acids in - are deletions.
